# Supplementary material for: Histidine dephosphorylation of the Gβ protein GPB‐1 promotes axon regeneration in C. elegans
Source: EMBO Rep. 2022 Oct 24;23(12):e55076. doi: 10.15252/embr.202255076 (PMC9724660; doi:10.15252/embr.202255076)
Supplement: Supplementary file 1 — Appendix [file EMBR-23-e55076-s001.pdf]

Appendix

Table of contents	Page
Appendix Table S1	2
Appendix Table S2	3
Appendix Table S3	4
Appendix Figure S1	5
Appendix Figure S2	7
Appendix Figure S3	8
Appendix Figure S4	9
Appendix Figure S5	11

Appendix Table S1. PHIP-1(H45A) binding proteins identified by yeast two-hybrid screen.

Gene	Gene product	Number of colonies
gpb-1	Gβ	1
gpd-2<br>*gpd-3*<br>*gpd-4*	GAPDH	1<br>12<br>8
unc-51	ULK homolog	1

Appendix Table S2. Strains used in this study.

Strain	Genotype
KU501	*juIs76 II*
KU96	*phip-1(km96) I; juIs76 II*
KU97	*juIs76 II; lhpp-1(km97) V*
KU98	*goa-1(km98) I; juIs76 II*
KU1461	*phip-1(km96) I; juIs76 II; kmEx1461 [Punc-25::phip-1]*
KU1462	*phip-1(km96) I; juIs76 II; kmEx1462 [Punc-25::phip-1(H45A)]*
KU1463	*juIs76 II; kmEx1463 [Punc-25::ndk-1]*
KU1464	*juIs76 II; kmEx1464 [Punc-25::ndk-1(H118N)]*
KU1465	*gpb-1(H266F) juIs76 II*
KU1466	*phip-1(km96) I; gpb-1(H266F) juIs76 II*
KU1467	*gpb-1(H266F) juIs76 II; kmEx1463 [Punc-25::ndk-1]*
KU1468	*goa-1(km98) phip-1(km96) I; juIs76 II*
KU455	*goa-1(Q205L) I; juIs76 II*
KU1469	*goa-1(Q205L) I; gpb-1(H266F) juIs76 II*
KU1343	*muIs32 II*
KU1470	*muIs32 II; unc-51(ks49) V*
KU1471	*phip-1(km96) I; muIs32 II*
KU1472	*phip-1(S112A) I; muIs32 II*
KU1473	*phip-1(S112E) I; muIs32 II*
KU1474	*phip-1(S112E) I; muIs32 II; unc-51(ks49) V*
KU1475	*gpb-1(H266F) muIs32 II; unc-51(ks49) V*
KU1476	*muIs32 II; lgg-2(tm6544) IV*
KU1477	*phip-1(km96) I; muIs32 II; lgg-2(tm6544) IV*
KU1478	*3XFLAG::gpb-1 juIs76 II*
KU1479	*phip-1(km96) I; 3XFLAG::gpb-1 juIs76 II*
KU1480	*phip-1(km96) I; 3XFLAG::gpb-1(H266F) juIs76 II*
KU1481	*juIs76 II; unc-51(ks49) V*

Appendix Table S3. DNA and RNA sequences.

phip-1(km96)	crispr RNA	gcucgcugacaucgccgaugguuuuagagcuaugcu
	PCR primer for genotyping (forward)	ttatcacagtgtgagagcattggg
	PCR primer for genotyping (reverse)	gaagataatgaaacaactgctctactc
lhpp-1(km97)	crispr RNA	aaacucccguuauaucgagcguuuuagagcuaugcu
	PCR primer for genotyping (forward)	catctaaacggaccctttctgcc
	PCR primer for genotyping (reverse)	gcacgcaaatgtttaccttgagg
goa-1(km98)	crispr RNA	cauggguuguaccaugucacguuuuagagcuaugcu
	PCR primer for genotyping (forward)	gagctgcaccacatacagtgagtg
	PCR primer for genotyping (reverse)	tacaatagtcgattttcctgattctcc
gpb-1(H266F)	crispr RNA	aauauuaucaugagaauacaguuuuagagcuaugcu
	ssDNA	gttcgacattcgtgctgatcaggaacttgcaatgtatt cttttgataatattatttgcggaatcactagt
	PCR primer for genotyping (forward)	tctccagacttccgcacattcatc
	PCR primer for genotyping (reverse)	ttatcgtgaccagccaatactcctg
phip-1(S112A)	crispr RNA	aacauucauuucucuaaugaguuuuagagcuaugcu
	ssDNA	cattttaaagcagaaatacccagattataatatccactt cgcgaacgacggatattgaatctccatgtttgagcatagtt
	PCR primer for genotyping (forward)	gtggaatccatgtttaattcccagtggaac
	PCR primer for genotyping (reverse)	gacgctccacaatgtacaatcgtc
phip-1(S112E)	crispr RNA	aacauucauuucucuaaugaguuuuagagcuaugcu
	ssDNA	cattttaaagcagaaatacccagattataatatccactt cgaaaacgacggatattgaatctccatgtttgagcatagtt
	PCR primer for genotyping (forward)	gtggaatccatgtttaattcccagtggaac
	PCR primer for genotyping (reverse)	gacgctccacaatgtacaatcgtc
3XFLAG::gpb-1	crispr RNA	aaguucgcucaucuugcugcguuuuagagcuaugcu
	ssDNA	cgtcgacacttccatcagtaccatcctccggagcacgcaccaccacc agcagcaagatggattacaaagaccatgatggtgactat aaggatcatgatattgactataaagacgatgacgataa gagcgaacttgaccaacttcgacaggaggctgaacag ctgaagtcgcagattcggg
	PCR primer for genotyping (forward)	aaaacgcgaacaccgaccaggagc
	PCR primer for genotyping (reverse)	attgctagaccatgctctggaacg

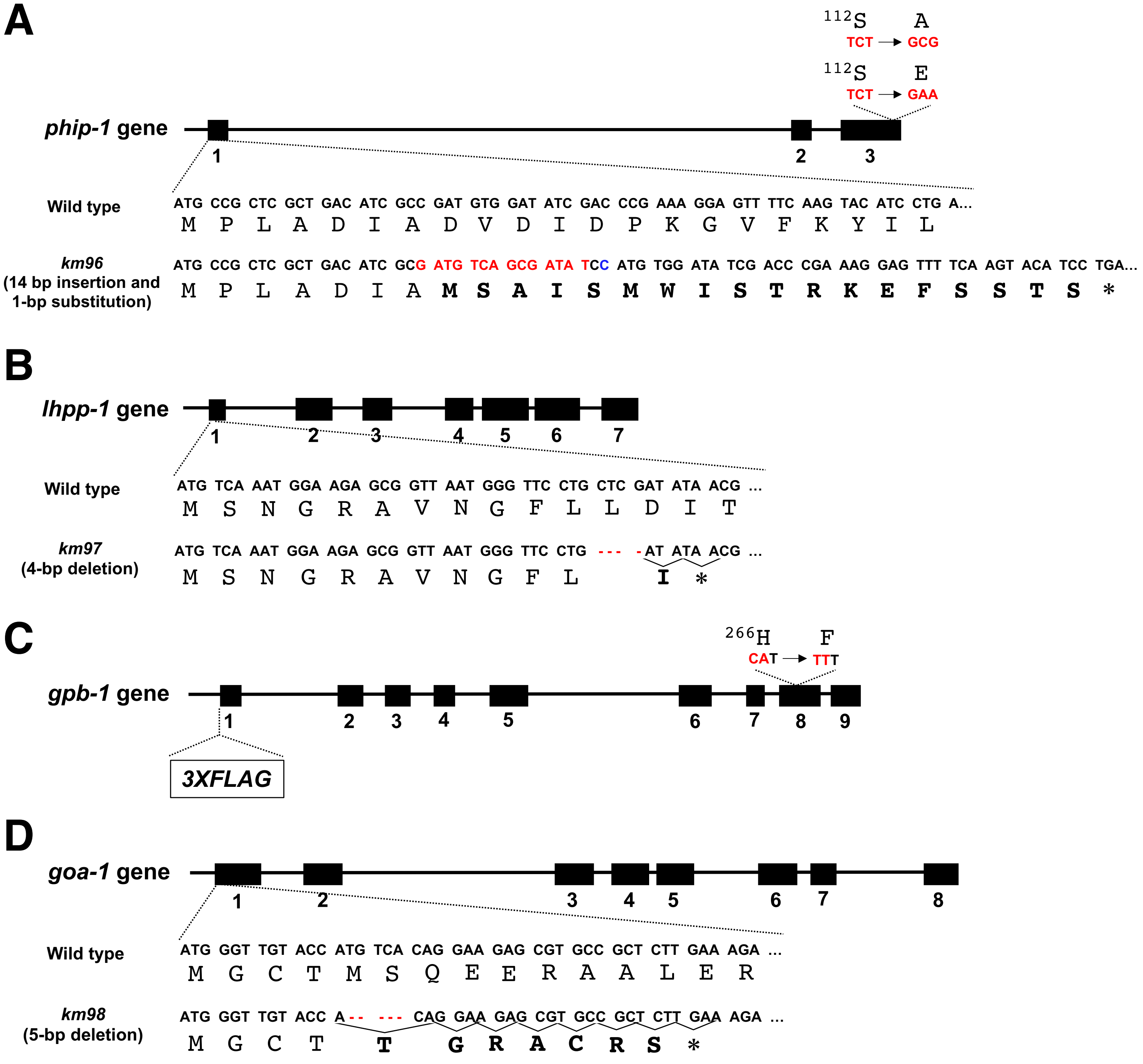

Appendix Figure S1. Genome editing of *phip-1*, *lhpp-1*, *gpb-1*, and *goa-1*.

A Genomic structure of the *phip-1* gene. Exons are indicated by boxes, while introns and untranslated regions are indicated by bars. The top and bottom letters indicate nucleotides and corresponding amino acids, respectively. The *phip-1(km96)* mutation is a 14 bp insertion (nucleotides in red) with 1-bp substitution (nucleotide in blue), which causes a frameshift (amino acids in bold) and premature stop codon (*) in exon 1. The *phip-1(S112A)* and *phip-1(S112E)* alleles are also shown.

B Genomic structure of the *lhpp-1* gene. The *lhpp-1(km97)* mutation is a 4-bp deletion, which causes a frameshift (amino acids in bold) and premature stop codon (*) in exon 1.

C Genomic structure of *gpb-1*. A 3XFLAG epitope tag was inserted into the N-terminus at the endogenous *gpb-1* locus with the CRISPR–Cas9 method. The *gpb-1(H266F)* allele is also shown.

D Genomic structure of the *goa-1* gene. The *goa-1(km98)* mutation is a 5-bp deletion, which causes a frameshift (amino acids in bold) and premature stop codon (*) in exon 1.

A

B

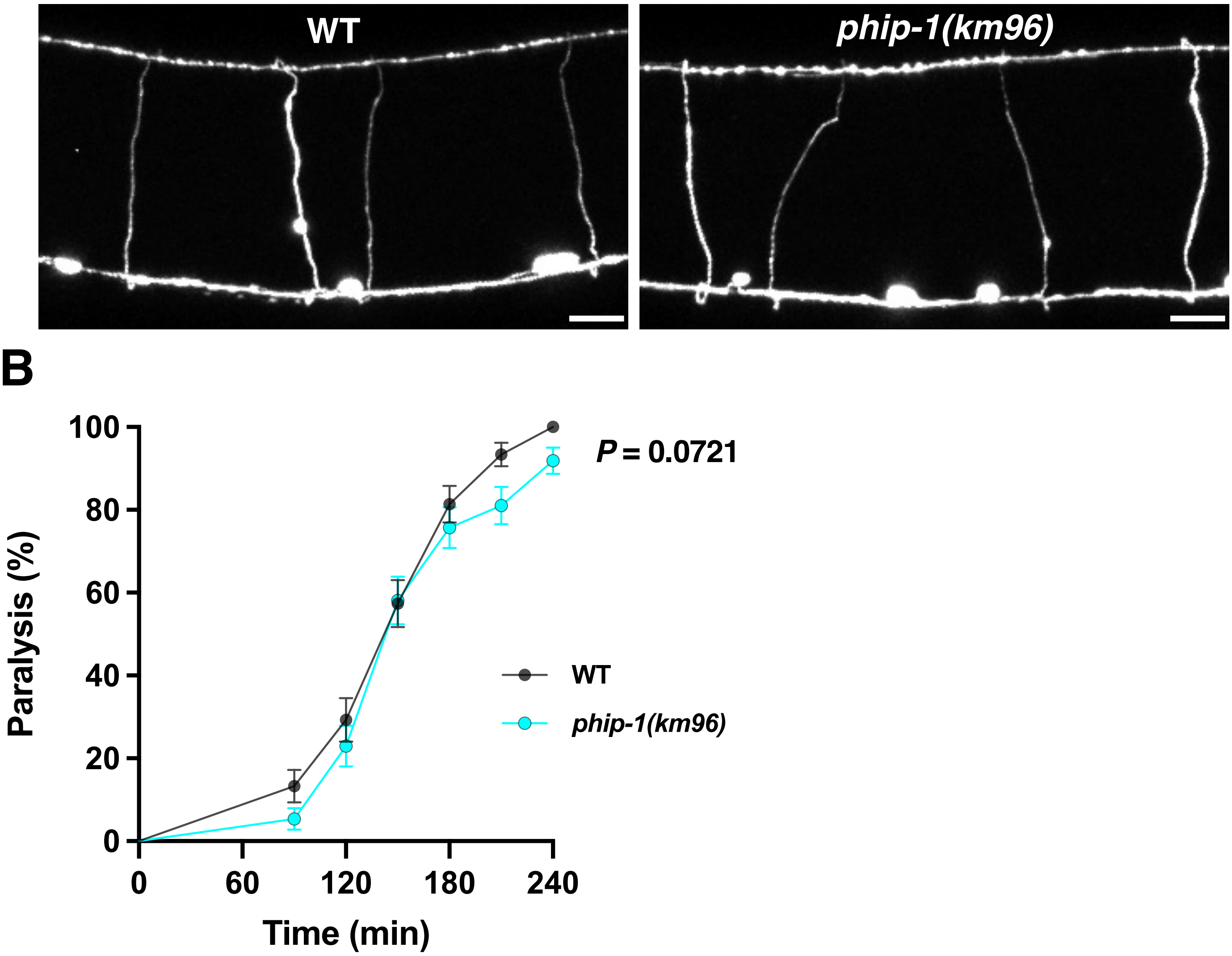

Appendix Figure S2. Effect of *phip-1* deletion on the morphology and function of D-type motor neurons.

A Morphology of D-type motor neurons. Fluorescent images of D-type motor neurons in wild-type and *phip-1(km96)* young adult animals carrying *Punc-25::gfp* are shown. D neurons are visualized by GFP under the control of the *unc-25* promoter. Scale bar, 10 μm.

B Aldicarb-induced paralysis assay. The rate of paralysis in wild-type and *phip-1(km96)* animals in the presence of 1 mM aldicarb are shown. Assays were performed blindly and in triplicate. Statistical significance was determined by the log-rank test. Error bars indicate SE.

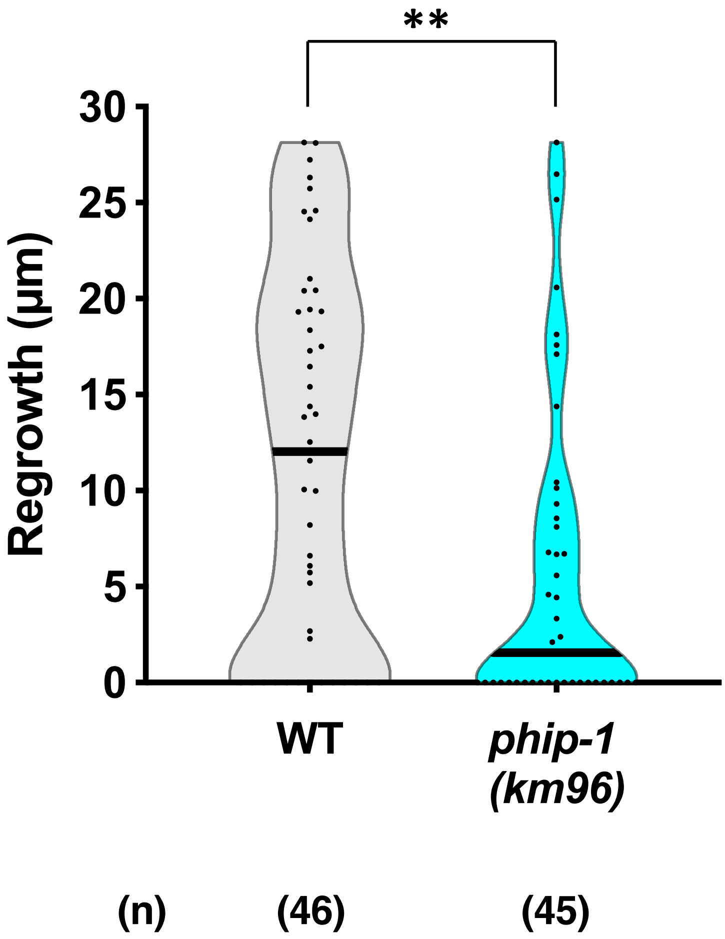

Appendix Figure S3. Effect of *phip-1* deletion on the length of D-type motor neurons after laser surgery.

Lengths of regenerating axons 24 h after laser surgery are shown. The number (n) of axons examined is indicated. The black bar in each violin plot indicates the median. **$P < 0.01$, as determined by the Mann–Whitney test.

A

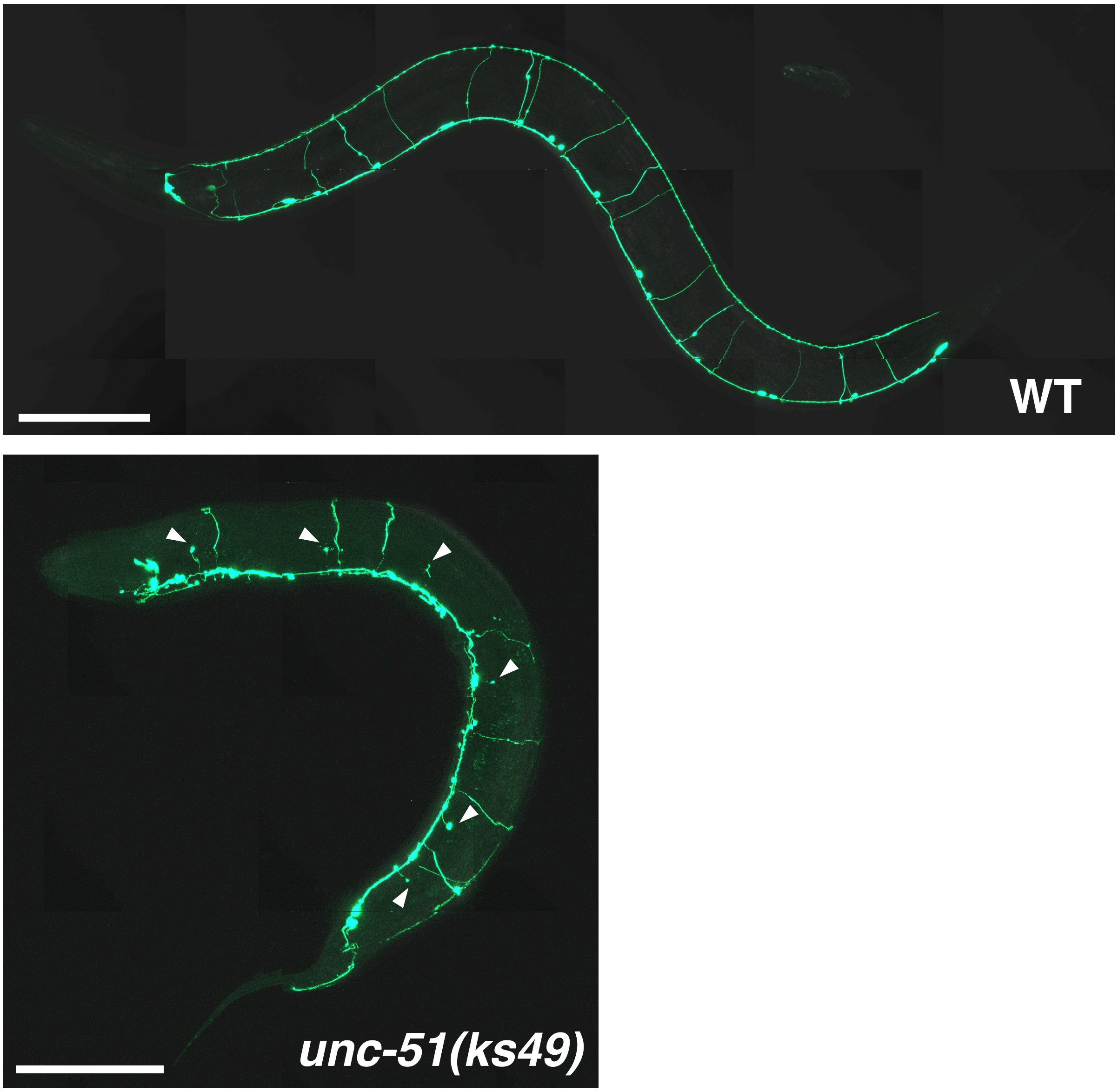

B

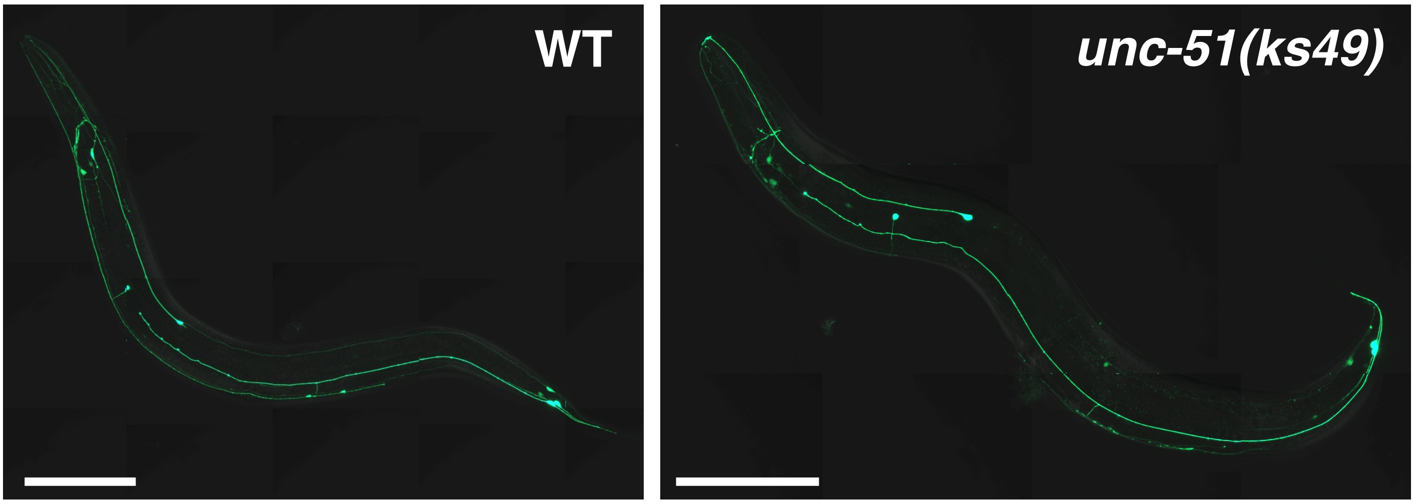

Appendix Figure S4. Effects of the *unc-51* mutation on the development of D-type motor neurons and PLM neurons.

A Morphology of D-type motor neurons. Fluorescent images of D-type motor neurons in wild-type and *unc-51(ks49)* young adult animals carrying *Punc-25::gfp* are shown. D neurons are visualized by GFP under the control of the *unc-25* promoter. White arrowheads indicate axons that failed to elongate from ventral to dorsal side during development. Scale bar, 100 μm.

B Morphology of PLM neurons. Fluorescent images of PLM neurons in wild-type and *unc-51(ks49)* young adult animals carrying *Pmec-7::gfp* are shown. PLM neurons are visualized by GFP under the control of the *mec-7* promoter. Scale bar, 100 μm.

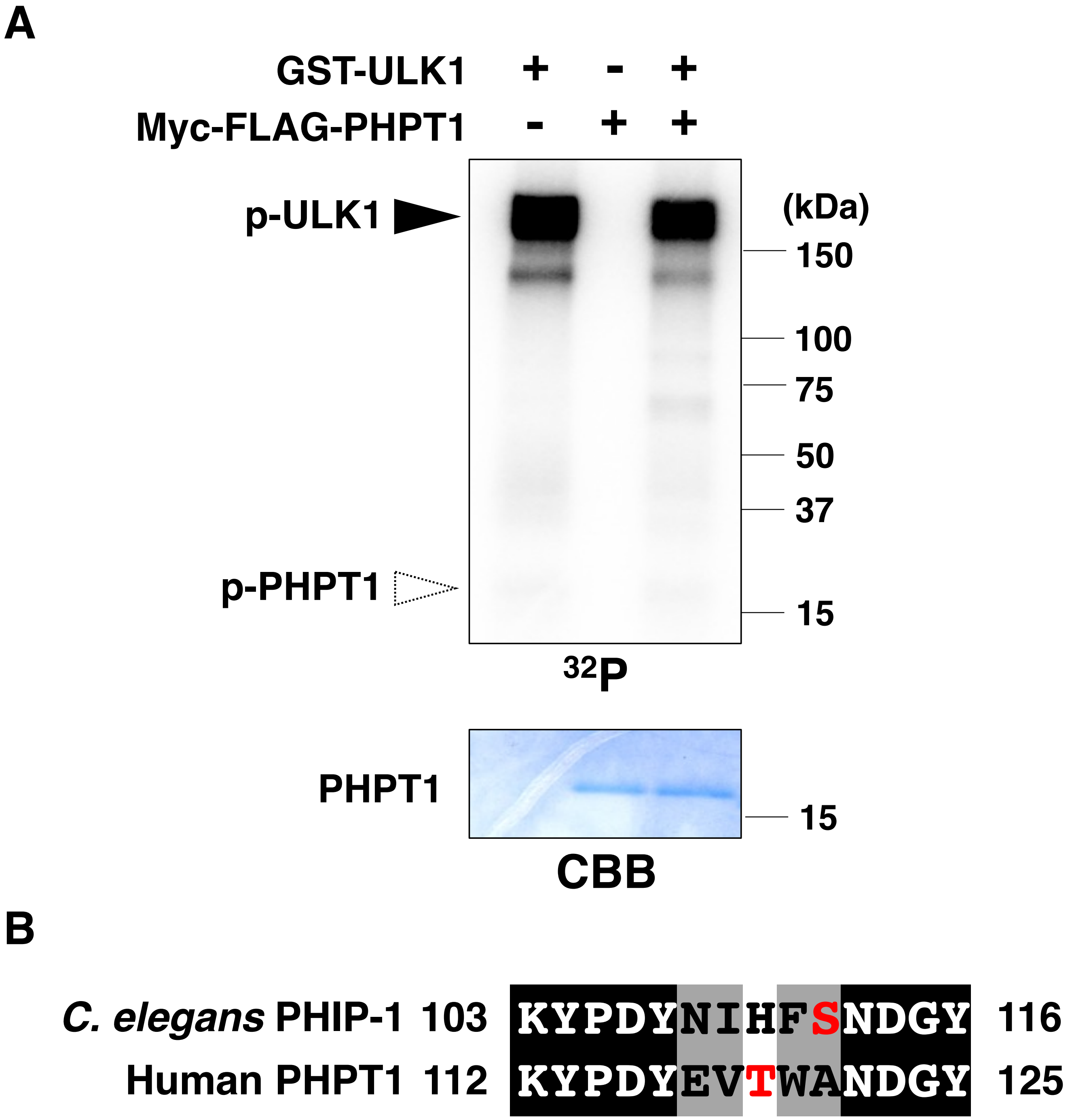

Appendix Figure S5. ULK1 does not phosphorylate PHPT1 in vitro.

A Phosphorylation of PHPT1 by ULK1. HEK293 cells were transfected with Myc-FLAG-PHPT1, and cell lysates were immunoprecipitated with anti-FLAG antibodies. Immunopurified PHPT1 was subjected to the in vitro kinase assay with recombinant GST-ULK1 and phosphorylation was detected by autoradiography. Protein input was confirmed by Coomassie Brilliant Blue (CBB) staining. Black arrowhead indicates ULK1 autophosphorylation.

B Sequence alignments of the C-terminal domain of human PHPT1 and *C. elegans* PHIP-1. Identical and similar residues are highlighted with black and gray shading, respectively. Ser-112 (in PHIP-1) and Thr-119 (in PHPT1) are shown.
