# Supplementary material for: Histidine dephosphorylation of the Gβ protein GPB‐1 promotes axon regeneration in C. elegans
Source: EMBO Rep. 2022 Oct 24;23(12):e55076. doi: 10.15252/embr.202255076 (PMC9724660; doi:10.15252/embr.202255076)
Supplement: Supplementary file 2 — Expanded View Figures PDF [file EMBR-23-e55076-s004.pdf]

Expanded View Figures

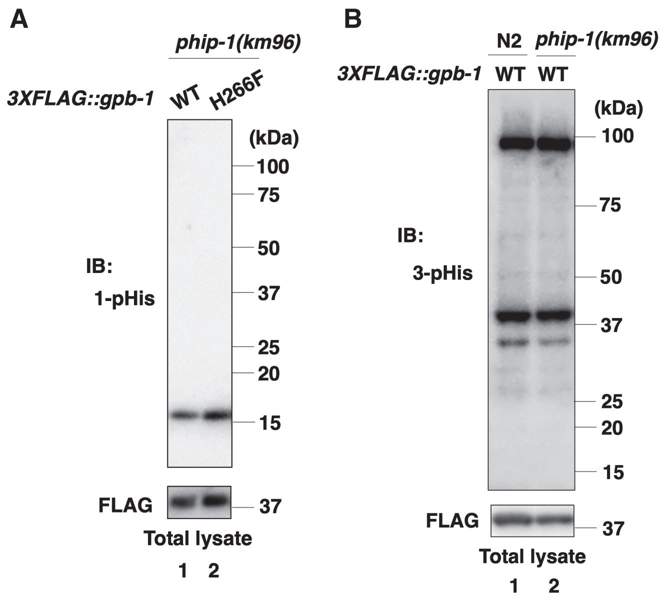

Figure EV1. His-phosphorylation in animals.

A 1-pHis levels in animals. The *phip-1(km96)* mutant animals carrying the *3XFLAG::gpb-1* or *3XFLAG::gpb-1(H266F)* knock-in allele were lysed. The lysates were immunoblotted (IB) with anti-1-pHis and anti-FLAG antibodies.

B The effect of the *phip-1(km96)* mutation on 3-pHis levels in animals. Wild-type N2 or *phip-1(km96)* mutant animals carrying the *3XFLAG::gpb-1* knock-in allele were lysed. The animal lysates were immunoblotted (IB) with anti-3-pHis and anti-FLAG antibodies.

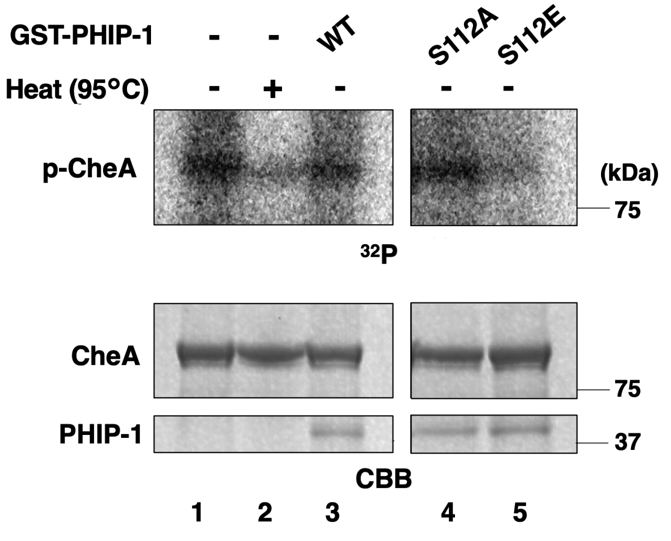

Figure EV2. Dephosphorylation of CheA by PHIP-1 *in vitro*.

GST-CheA was first incubated without GST-PHIP-1 for autophosphorylation. A-utophosphorylated CheA was then equally aliquoted and subjected to the *in vitro* phosphatase assay with GST-PHIP-1 or its variants. Phosphorylated CheA was detected by autoradiography. A heated sample (95°C) was used as a negative control. Protein input was confirmed by Coomassie Brilliant Blue (CBB) staining.

© 2022 The Authors

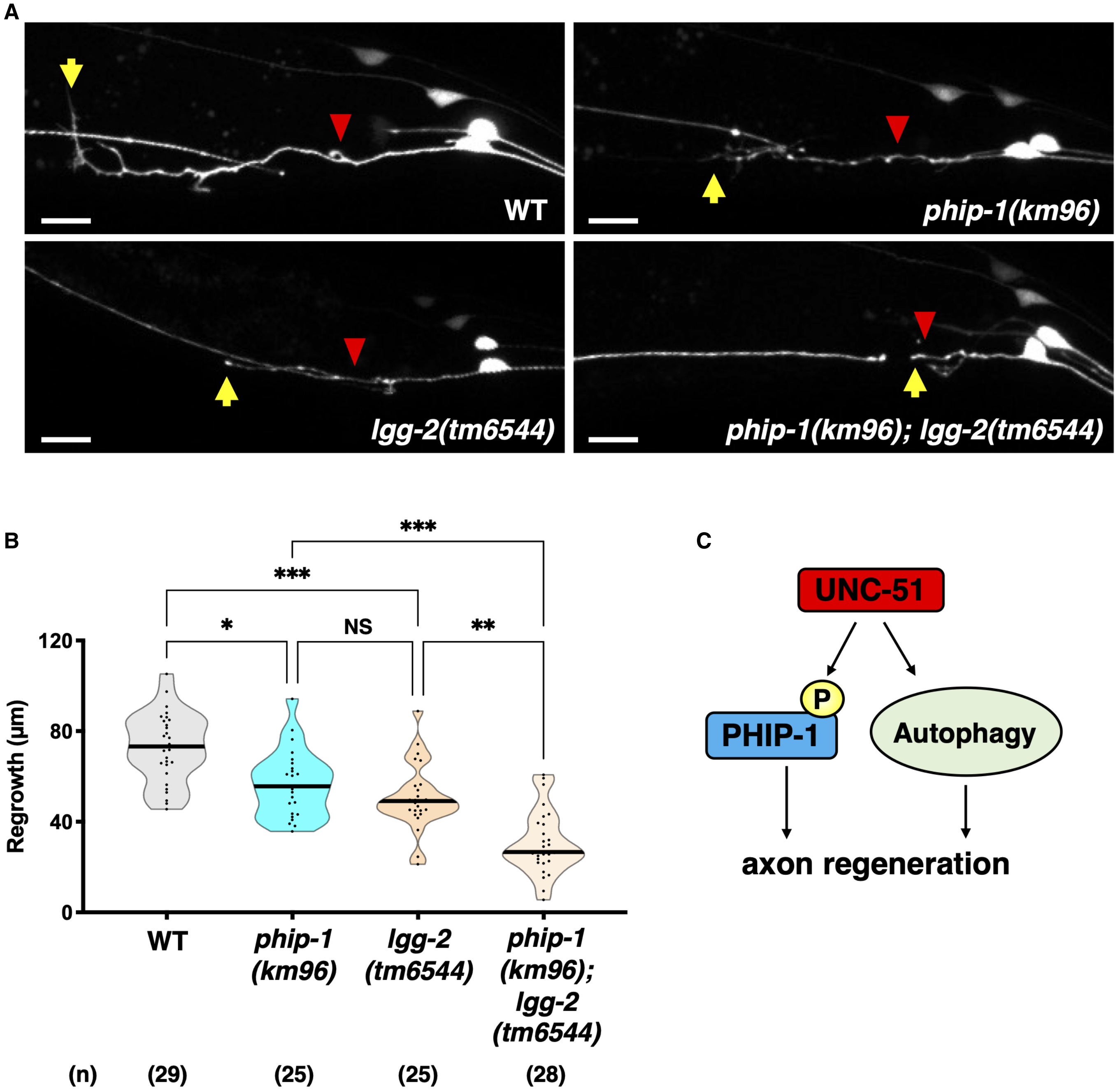

Figure EV3. UNC-51 regulates axon regeneration via PHIP-1 and autophagy.

A Representative PLM sensory neurons in indicated genotypes 24 h after laser surgery. Red arrowheads indicate cut sites. Yellow arrows indicate the tip of axotomized axons. Scale bar, 10 µm.

B Length of PLM regrowth 24 h after laser surgery. The number (*n*) of axons examined from two biological replicates is indicated. The black bar in each violin plot indicates the median. $^*P < 0.05$, $^{**}P < 0.01$, $^{***}P < 0.001$, as determined by the Kruskal–Wallis test and Dunn's multiple comparison test. NS, not significant.

C Downstream targets of UNC-51. UNC-51 promotes axon regeneration via phosphorylation of PHIP-1 and autophagy.

© 2022 The Authors
