# Supplementary material for: Histidine dephosphorylation of the Gβ protein GPB‐1 promotes axon regeneration in C. elegans
Source: EMBO Rep. 2022 Oct 24;23(12):e55076. doi: 10.15252/embr.202255076 (PMC9724660; doi:10.15252/embr.202255076)
Supplement: Supplementary file 3 — PDF+ [file EMBR-23-e55076-s003.pdf]

Article

TRANSPARENT PROCESS
OPEN ACCESS

EMBO reports

Histidine dephosphorylation of the Gβ protein GPB-1 promotes axon regeneration in *C. elegans*

Yoshiki Sakai, Hiroshi Hanafusa, Naoki Hisamoto* & Kunihiro Matsumoto**

Abstract

Histidine phosphorylation is an emerging noncanonical protein phosphorylation in animals, yet its physiological role remains largely unexplored. The protein histidine phosphatase (PHPT1) was recently identified for the first time in mammals. Here, we report that PHIP-1, an ortholog of PHPT1 in *Caenorhabditis elegans*, promotes axon regeneration by dephosphorylating GPB-1 Gβ at His-266 and inactivating GOA-1 Goα signaling, a negative regulator of axon regeneration. Overexpression of the histidine kinase NDK-1 also inhibits axon regeneration via GPB-1 His-266 phosphorylation. Thus, His-phosphorylation plays an antiregenerative role in *C. elegans*. Furthermore, we identify a conserved UNC-51/ULK kinase that functions in autophagy as a PHIP-1-binding protein. We demonstrate that UNC-51 phosphorylates PHIP-1 at Ser-112 and activates its catalytic activity and that this phosphorylation is required for PHIP-1-mediated axon regeneration. This study reveals a molecular link from ULK to protein histidine phosphatase, which facilitates axon regeneration by inhibiting trimeric G protein signaling.

Keywords axon regeneration; *C. elegans*; Gβ; His-phosphorylation; ULK
Subject Categories Neuroscience; Post-translational Modifications & Proteolysis
DOI 10.15252/embr.202255076 | Received 18 March 2022 | Revised 30 September 2022 | Accepted 4 October 2022 | Published online 24 October 2022
EMBO Reports (2022) 23: e55076

Introduction

Protein phosphorylation is one of the most important post-translational modifications that regulate and diversify the function of proteins. Although the phosphorylation of serine, threonine, and tyrosine are well characterized, relatively little is known about the phosphorylation of histidine (pHis). In prokaryotes and lower eukaryotes, such as yeast, fungi, and plants, two or multicomponent signaling systems have been discovered, in which protein His-kinases are crucial mediators of cellular responses, including bacterial chemotaxis and other sensing systems (Hess *et al*, 1988; Swanson *et al*, 1994). Recently, His-phosphorylation has been attracting attention as a new form of protein phosphorylation in mammals (Fuhs & Hunter, 2017; Lu & Hunter, 2018). Currently, mammals contain His-kinases, nucleoside diphosphate kinases (NDPKs; Hartsough *et al*, 2002; Hippe *et al*, 2003), as well as three His-phosphatases, protein histidine phosphatase 1 (PHPT1; Ek *et al*, 2002), phospholysine phosphohistidine inorganic pyrophosphate phosphatase (LHPP; Hindupur *et al*, 2018), and phosphoglycerate mutase 5 (PGAM5; Panda *et al*, 2016). Recent biochemical and genetic experiments revealed that NDPK phosphorylates the heterotrimeric G protein β subunit (Gβ; Cuello *et al*, 2003; Hippe *et al*, 2003), the Ca^{2+}-activated K^{+} channel Kca3.1 (Srivastava *et al*, 2006), and the transient receptor potential-vanilloid-5 (TRPV5; Cai *et al*, 2014) and affects the function of these proteins (Cuello *et al*, 2003; Srivastava *et al*, 2006, 2008, 2016; Cai *et al*, 2014). These findings provided definitive proof that His-phosphorylation is important in mammalian biology.

Signaling through G protein activation is the most widely used signaling pathway. Various G protein-coupled receptors (GPCRs) transmit extracellular signals via heterotrimeric G proteins composed of Gα, Gβ, and Gγ (Oldham & Hamm, 2008). The ligand-bound GPCR acts as a guanine-nucleotide exchange factor (GEF) by facilitating the exchange of guanosine diphosphate (GDP) for guanosine triphosphate (GTP) on Gα. Gα dissociates from Gβγ upon GTP binding. Both GTP-bound Gα and free Gβγ regulate downstream effectors to elicit cellular responses. The complexity of the trimeric G protein signaling network is increased by the existence of accessory proteins, such as nonreceptor GEFs that regulate the activity of Gα proteins (Afshar *et al*, 2004; Hess *et al*, 2004). The nonreceptor GEF mimics GPCR action; however, it is a cytoplasmic factor rather than a membrane receptor. Furthermore, His-phosphorylation of Gβ by NDPK is involved in the G protein activation. The high-energy pHis intermediate is transferred to GDP liganded to Gα, generating a GTP-bound form, which results in receptor-independent G protein activation (Cuello *et al*, 2003; Hippe *et al*, 2003). However, the precise implication of NDPK-mediated Gβ phosphorylation on trimeric G protein signaling *in vivo* remains elusive.

The nematode *Caenorhabditis elegans* is a tractable genetic model. The *C. elegans* genome encodes only single orthologs of mammalian NDPK and PHPT1, NDK-1 and PHIP-1, respectively. In this study, we investigated the role of protein His-phosphorylation in *C. elegans*. We discovered that *ndk-1* overexpression or *phip-1* inactivation inhibits axon regeneration mediated through His-266

Division of Biological Science, Graduate School of Science, Nagoya University, Nagoya, Japan
*Corresponding author. Tel: +81 52 789 3000; E-mail: i45556a@cc.nagoya-u.ac.jp
**Corresponding author. Tel: +81 52 789 2589; E-mail: g44177a@nucc.cc.nagoya-u.ac.jp

© 2022 The Authors. Published under the terms of the CC BY 4.0 license

phosphorylation of GPB-1 Gβ. Furthermore, we identified UNC-51, a conserved serine/threonine protein kinase with homology to human ULK, which is required for autophagy, as a binding protein for PHIP-1 (Wang & Kundu, 2017). Here, we demonstrate that UNC-51 phosphorylates PHIP-1 at the Ser-112, thereby activating the catalytic activity of PHIP-1 and promoting axon regeneration through pHis-dephosphorylation of GPB-1. Our findings reveal a molecular link from UNC-51/ULK kinase to protein His-phosphatase for the regulation of axon regeneration by modulating trimeric G protein signaling.

Results

PHIP-1 is required for axon regeneration

Protein phosphorylation is regulated by kinases on the one hand and by phosphatases on the other. In mammals, His-phosphorylation is regulated by NDPK, a His-kinase, and PHPT1, a pHis-phosphatase (Fuhs & Hunter, 2017; Lu & Hunter, 2018; Fig 1A). *Caenorhabditis elegans* contains these orthologs, NDK-1 and PHIP-1 (Klumpp *et al*, 2002; Masoudi *et al*, 2013; Fig 1B). To elucidate the role of His-phosphorylation in *C. elegans*, we performed a genetic analysis of NDK-1 and PHIP-1. NDK-1 has a housekeeping function, which regulates nucleotide homeostasis, and its loss can be lethal (Masoudi *et al*, 2013). Therefore, we focused on the *phip-1* gene. Using the CRISPR–Cas9 system, we generated a null mutant of the *phip-1* gene, *phip-1(km96)* (Fig 1B and Appendix Fig S1A). In contrast to *ndk-1* deletion, *phip-1* deletion does not cause any obvious phenotypic alterations, and it is indistinguishable from wild-type animals.

PHIP-1 is exclusively localized in motor neurons along the ventral nerve cord (Klumpp *et al*, 2002). However, the *phip-1(km96)* mutation had no obvious effect on nerve development or synaptic function in motor neurons (Appendix Fig S2A and B). Next, we examined whether PHIP-1 affects axon regeneration by evaluating regrowth after laser axotomy in γ-aminobutyric acid (GABA)-releasing D-type motor neurons (Fig 2A). We found that axon regeneration was significantly reduced in *phip-1(km96)* mutants (Fig 2B and C). The length of regenerated axons in *phip-1(km96)* mutants was shorter than that in wild-type animals (Appendix Fig S3). Thus, PHIP-1 is required for efficient axon regeneration after injury. To examine whether PHIP-1 can function cell-autonomously, we specifically expressed the *phip-1* cDNA by the *unc-25* promoter in GABAergic neurons of *phip-1* mutants. As a result, *phip-1* expression in D-type motor neurons rescued the axon regeneration defect of *phip-1(km96)* mutants (Fig 2C), implying that PHIP-1 functions cell-autonomously in injured neurons. The His-53 residue of mammalian PHPT1 is essential for its phosphatase activity (Busam *et al*, 2006; Fig 1B). Accordingly, the H53A mutant, in which His-53 is replaced with alanine, is defective in pHis-phosphatase activity. Similar to mammalian PHPT1, PHIP-1 possesses a conserved site, His-45, corresponding to mammalian His-53 (Fig 1B). We generated a mutant of PHIP-1 [PHIP-1(H45A)] with His-45 mutated to alanine to determine the significance of PHIP-1 pHis-phosphatase activity in axon regeneration. We found that H45A point mutation could not rescue the *phip-1(km96)* phenotype (Fig 2C). These findings indicate that PHIP-1 is essential for axon regeneration in a manner dependent on its pHis-phosphatase activity.

LHPP has recently been identified as a second pHis-phosphatase in mammalian cells (Hindupur *et al*, 2018). Since *C. elegans* also contains this ortholog LHPP-1 (Fig 1B), we used the CRISPR–Cas9 system to construct a null mutant, *lhpp-1(km97)* (Fig 1B and Appendix Fig S1B). In contrast to *phip-1* deletion, *lhpp-1(km97)* mutation did not affect axon regeneration (Fig 2C). These results indicate that PHIP-1 is specifically required for axon regeneration.

NDK-1 overexpression inhibits axon regeneration

Since NDK-1 is predicted to function as a protein His-kinase (Fig 1A), we examined if NDK-1 would also participate in axon regeneration. We found that wild-type *ndk-1* overexpression in GABAergic neurons of wild-type animals significantly reduced axon regeneration after laser injury (Fig 2B and D). The kinase activity of NDPK requires autophosphorylation at the catalytic His-118 residue (Lecroisey *et al*, 1995), which is also conserved in NDK-1 (Fig 1B). In addition, overexpression of the kinase-dead *ndk-1(H118N)* containing His-118 substitution for asparagine (H118N) (Fig 1B) did not inhibit axon regeneration (Fig 2D), indicating that kinase activity is required for NDK-1 inhibitory effect. Furthermore, when *ndk-1* was overexpressed in *phip-1(km96)* mutants, we observed that the impaired regeneration was no greater than that observed in the *phip-1(km96)* mutant (Fig 2D), suggesting that NDK-1 and PHIP-1 act in the same pathway. These results indicate that NDK-1 and PHIP-1 regulate axon regeneration through His-phosphorylation of the same target protein.

NDK-1 and PHIP-1 regulate axon regeneration through His-phosphorylation of the Gβ subunit GPB-1

To identify the targets for PHIP-1, we performed a yeast two-hybrid screen using phosphatase-negative PHIP-1(H45A) (Fig 1B) as bait. We identified five genes: *gpb-1*, *gpd-2/gpd-3/gpd-4*, and *unc-51*, which encode Gβ, glyceraldehyde-3-phosphate dehydrogenase (GAPDH), and a homolog of ULK kinase, respectively (Fig 3A and Appendix Table S1). Interestingly, mammalian GNB1 Gβ and GAPDH are pHis proteins (Fuhs *et al*, 2015). These results suggest that GPB-1 and GPD-2/GPD-3/GPD-4 are candidate targets for PHIP-1. Here, we focused on the Gβ subunit GPB-1.

In mammals, NDPK has been shown to phosphorylate GNB1 Gβ at His-266 and participate in trimeric G protein activation (Cuello *et al*, 2003; Hippe *et al*, 2003). *Caenorhabditis elegans* expresses two Gβ subunits, GPB-1 and GPB-2 (van der Voorn *et al*, 1990; Robatzek *et al*, 2001). The His-266 phosphorylation site is conserved in GPB-1, but not in GPB-2 (Fig 3B). Therefore, we genetically examined whether pHis-266 in GPB-1 is involved in axon regeneration regulated by NDK-1 and PHIP-1. We engineered the *gpb-1(H266F)* mutation by replacing the codon encoding the His-266 residue with the phenylalanine codon in the endogenous *gpb-1* locus using the CRISPR–Cas9 mutagenesis (Appendix Fig S1C). As a result, we found that *gpb-1(H266F)* mutation could suppress the defect in axon regeneration caused by *ndk-1* overexpression or the *phip-1(km96)* deletion (Fig 3C). These results confirm the possibility that NDK-1 and PHIP-1 regulate axon regeneration by phosphorylating GPB-1 at His-266.

 © 2022 The Authors

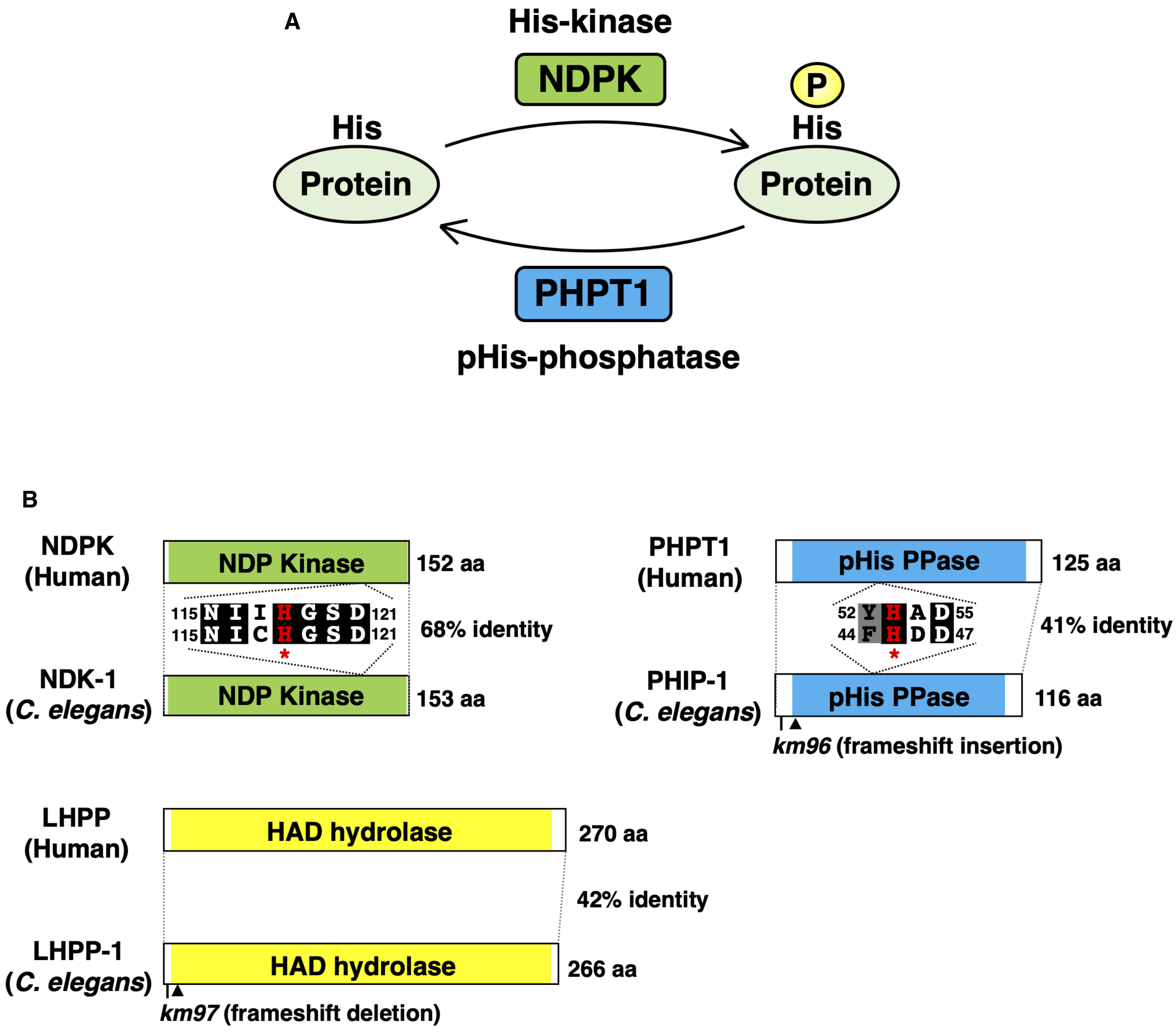

Figure 1. His-kinase and pHis-phosphatase.

A Protein His-phosphorylation is regulated by His-kinase and pHis-phosphatase.

B NDK-1, PHIP-1, and LHPP-1 structures. Schematic diagrams of NDK-1, PHIP-1, LHPP-1, and their mammalian counterparts are shown. The NDP kinase domain is shown in green and the phosphohistidine (pHis) phosphatase domain in blue. Kinase-dead NDK-1(H118N) and catalytically inactive PHIP-1(H45A) mutations are denoted by asterisks. Identical and similar residues are highlighted with black and gray shading, respectively. Arrowheads indicate premature stop codons caused by *km96* and *km97* mutations.

His-phosphorylation of GPB-1

Next, we investigated His-phosphorylation of GPB-1 in *C. elegans*. pHis exists as two isomers, 1-pHis and 3-pHis, depending on the position of the phospho-acceptor nitrogen in the imidazole ring of histidine at positions, N1 and N3, respectively (Fuhs *et al*, 2015). Because the phosphoramidate (P–N) bond in pHis is thermally unstable, detecting pHis in biological samples is challenging. This problem has been largely solved with the development of monoclonal antibodies that specifically recognize 1-pHis or 3-pHis (Fuhs *et al*, 2015). We used these antibodies to evaluate His-phosphorylation in animals. To detect GPB-1, we tagged endogenous GPB-1 with 3XFLAG using CRISPR–Cas9-mediated genome editing (Appendix Fig S1C).

Immunoblot signals for 1-pHis and 3-pHis detected one 1-pHis- and two- or three 3-pHis-positive proteins in total lysates of *phip-1 (km96)* mutant animals (Fig 4A, lane 1 and Fig EV1A and B). The 17-kDa band observed with the 1-pHis antibody is likely to be NDK-1 (Fig EV1A). Indeed, mammalian NDPK autophosphorylates at position N1 of His-118 (Lecroisey *et al*, 1995), which is also

© 2022 The Authors

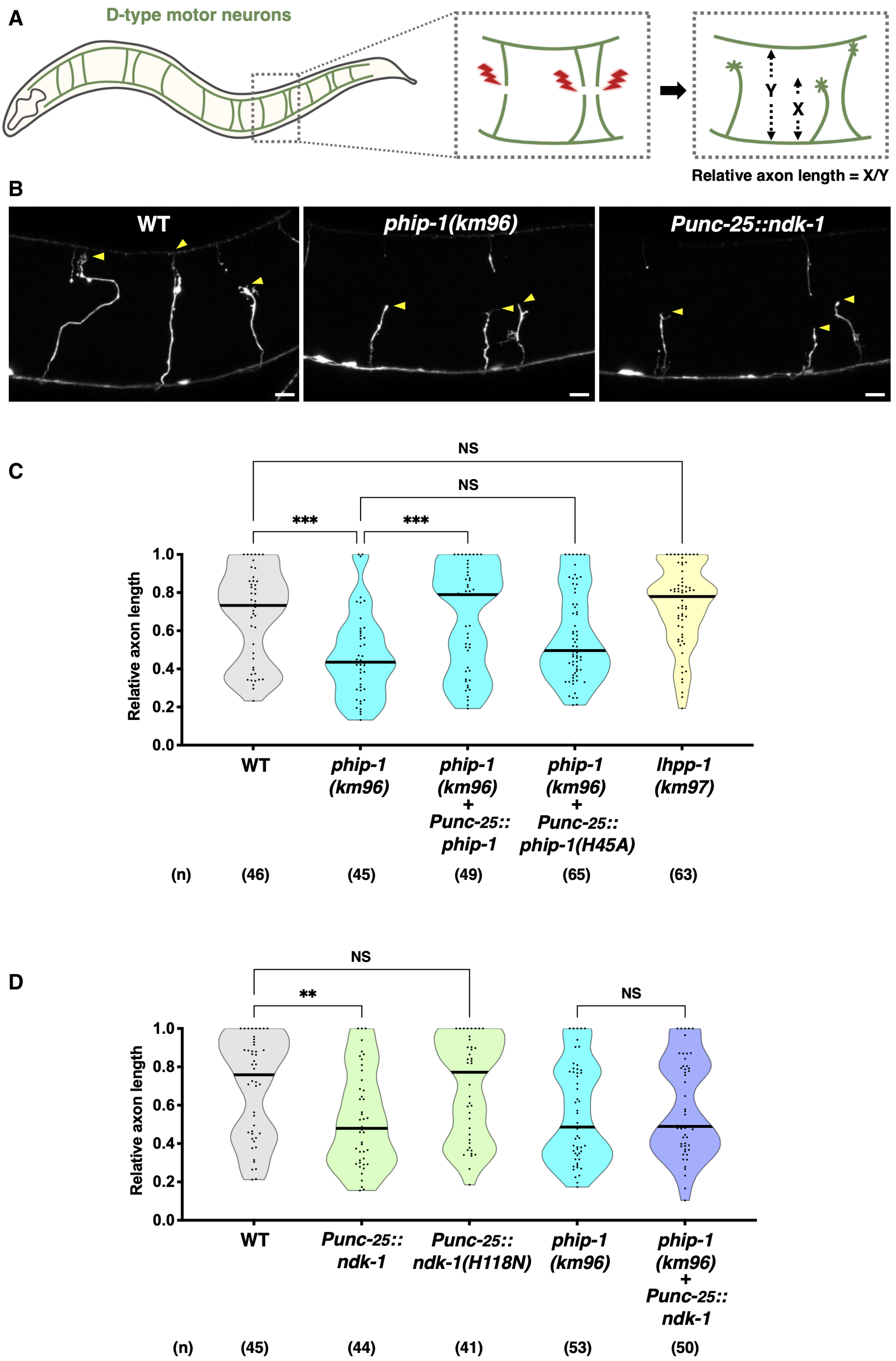

Figure 2.

 © 2022 The Authors

Figure 2. Protein His-phosphorylation inhibits axon regeneration.

A Scheme for axotomy and relative axon length measurements of GABAergic D-type motor neurons in *Caenorhabditis elegans*. Relative axon length was determined by the distance from the ventral nerve cord to the injured axon tip (X) normalized by the distance from the ventral nerve cord to the dorsal nerve cord (Y).

B Representative D-type motor neurons in wild-type animals, *phip-1(km96)* mutants, and NDK-1-overexpressing animals 24 h after laser surgery. Arrowheads indicate the tip of axotomized axons. Scale bar, 10 μm.

C, D Relative axon length 24 h after laser surgery at the young adult stage. The number (*n*) of axons examined from three biological replicates is indicated. The black bar in each violin plot indicates the median. $^{**}P < 0.01$, $^{***}P < 0.001$, as determined by the Kruskal–Wallis test and Dunn's multiple comparison test. NS, not significant.

conserved in NDK-1 (Fig 1B). When the lysate, dissolved in sample buffer, was heated at 95°C for 15 min prior to SDS–PAGE, the 3-pHis bands disappeared (Fig 4A, lane 2), indicating that the signals detected in the unheated sample are indeed 3-pHis proteins. Based on molecular weight analysis, the low molecular weight (37 kDa) 3-pHis protein corresponds to 3XFLAG::GPB-1. Consistently, the intensity of the 3-pHis signal was reduced in *phip-1(km96)* mutants expressing the nonphosphorylatable FLAG::GPB-1(H266F) mutant protein (Fig 4A, lane 3 and Appendix Fig S1C). This result also suggests that GPB-1 has additional pHis site(s). Thus, PHIP-1 dephosphorylates GPB-1 pHis-266 in animals. However, the intensity of the 3-pHis signals did not increase in *phip-1(km96)* mutants compared with wild-type animals (Fig EV1B), suggesting that under normal conditions, PHIP-1 is an inactive pHis-phosphatase.

To determine whether NDK-1 phosphorylates GPB-1 at His-266, an *in vitro* kinase assay was performed using purified recombinant glutathione *S*-transferase (GST)-tagged NDK-1. In mammalian cells, NDPK forms a complex with Gβγ and acts as a His-kinase for Gβ (Cuello *et al*, 2003; Hippe *et al*, 2003; Wieland *et al*, 2010). Therefore, we used the *C. elegans* Gβγ complex as a substrate. Because *C. elegans* has two Gγ subunits, namely GPC-1 and GPC-2 (Jansen *et al*, 2002), and GPC-2 works with GPB-1 in *C. elegans* (Gotta & Ahringer, 2001), we used GPC-2 for the *in vitro* kinase assay. We co-expressed HA-tagged GPB-1 and T7-tagged GPC-2 in mammalian COS-7 cells. The GPB-1–GPC-2 complex was then immunopurified with anti-HA antibodies and incubated with GST-NDK-1 *in vitro*. GPB-1 phosphorylation was detected with anti-3-pHis antibodies. We found that NDK-1 phosphorylated GPB-1 and that the phosphorylation of GPB-1(H266F) by NDK-1 was reduced but not eliminated (Fig 4B, lane 1–4). These results support the possibility that NDK-1 phosphorylates multiple His-sites, including His-266, in GPB-1. Next, we tested whether PHIP-1 dephosphorylates pHis-GPB-1 *in vitro* using recombinant GST-tagged PHIP-1. We found that wild-type PHIP-1 dephosphorylated pHis-GPB-1; however, phosphatase-negative PHIP-1(H45A) did not (Fig 4C, lane 1–3). Taken together, these results indicate that NDK-1 and PHIP-1 phosphorylate and dephosphorylate GPB-1 at His residues, respectively.

GPB-1 His-phosphorylation suppresses axon regeneration by activating GOA-1 Goα

When NDPK phosphorylates mammalian GNB1 Gβ His-266, the high-energy pHis intermediate is assumed to be transferred to Gα-GDP, resulting in the generation of Gα-GTP independent of GPCR (Cuello *et al*, 2003; Hippe *et al*, 2003; Fig 5A). We previously demonstrated that active GOA-1 Goα acts as a negative regulator of axon regeneration (Pastuhov *et al*, 2012), implying that GOA-1 is a candidate for Gα activated by His-phosphorylation of GPB-1 Gβ in the regulation of axon regeneration (Fig 5A). Therefore, we examined the genetic interaction between *phip-1* and *goa-1* and found that *goa-1(km98)* null mutation (Appendix Fig S1D) could suppress the regeneration defect caused by *phip-1(km96)* mutation (Fig 5B). This result indicates that GOA-1 functions downstream of GPB-1 His-phosphorylation. Furthermore, the *gpb-1(H266F)* mutation did not suppress axon regeneration defect by the gain-of-function *goa-1 (Q205L)* mutation (Fig 5B). These findings reveal that that GPB-1 His-phosphorylation inhibits axon regeneration by activating GOA-1.

In *C. elegans*, two different Gα subunits, namely EGL-30 Gqα and GOA-1 Goα participate in axon regeneration (Pastuhov *et al*, 2012; Fig 5C). EGL-30 activates phospholipase Cβ (PLCβ) EGL-8, which in turn generates DAG, resulting in the activation of the protein kinase C (PKC) homolog TPA-1. TPA-1 promotes axon regeneration by activating the JNK MAP kinase (MAPK) cascade. GTP-bound GOA-1 antagonizes the EGL-30 signaling cascade and inhibits axon regeneration. This inhibition is mediated by His-266 phosphorylation of GPB-1 Gβ, which leads to activation of GOA-1 Goα signaling. Thus, these findings suggest a link between the GPCR-independent activation of Goα and His-phosphorylation of Gβ in the regulation of axon regeneration (Fig 5C).

UNC-51 phosphorylates PHIP-1 at Ser-112

To initiate axon regeneration after axon injury, GOA-1 activation by GPB-1 His-phosphorylation must be downregulated. How does axon injury cause pHis-dephosphorylation of GPB-1? Axon injury may inhibit the kinase activity of NDK-1 or activate PHIP-1 pHis-

Figure 3. NDK-1 and PHIP-1 regulate axon regeneration through His-phosphorylation of the Gβ subunit GPB-1.

A PHIP-1 interaction with GPB-1 by yeast two-hybrid assay. The reporter strain PJ69-4A was co-transformed with expression vectors encoding GAL4 DBD-PHIP-1(H45A) and GAL4 AD-GPB-1, as indicated. Yeast strains carrying the indicated plasmids were cultured on a selective plate lacking histidine and containing 5 mM 5-aminotriazole for 4 days.

B His-phosphorylation site in Gβ. Sequence alignments of the His-phosphorylation site and flanking amino acids among human GNB1, GPB-1, and GPB-2 are shown. Identical and similar residues are highlighted with black and gray shading, respectively. The His-phosphorylation site, His-266, is indicated by an arrowhead.

C Relative axon length 24 h after laser surgery at the young adult stage. The number (*n*) of axons examined from three biological replicates is indicated. The black bar in each violin plot indicates the median. $^{**}P < 0.01$, $^{***}P < 0.001$, as determined by the Kruskal–Wallis test and Dunn's multiple comparison test. NS, not significant.

© 2022 The Authors

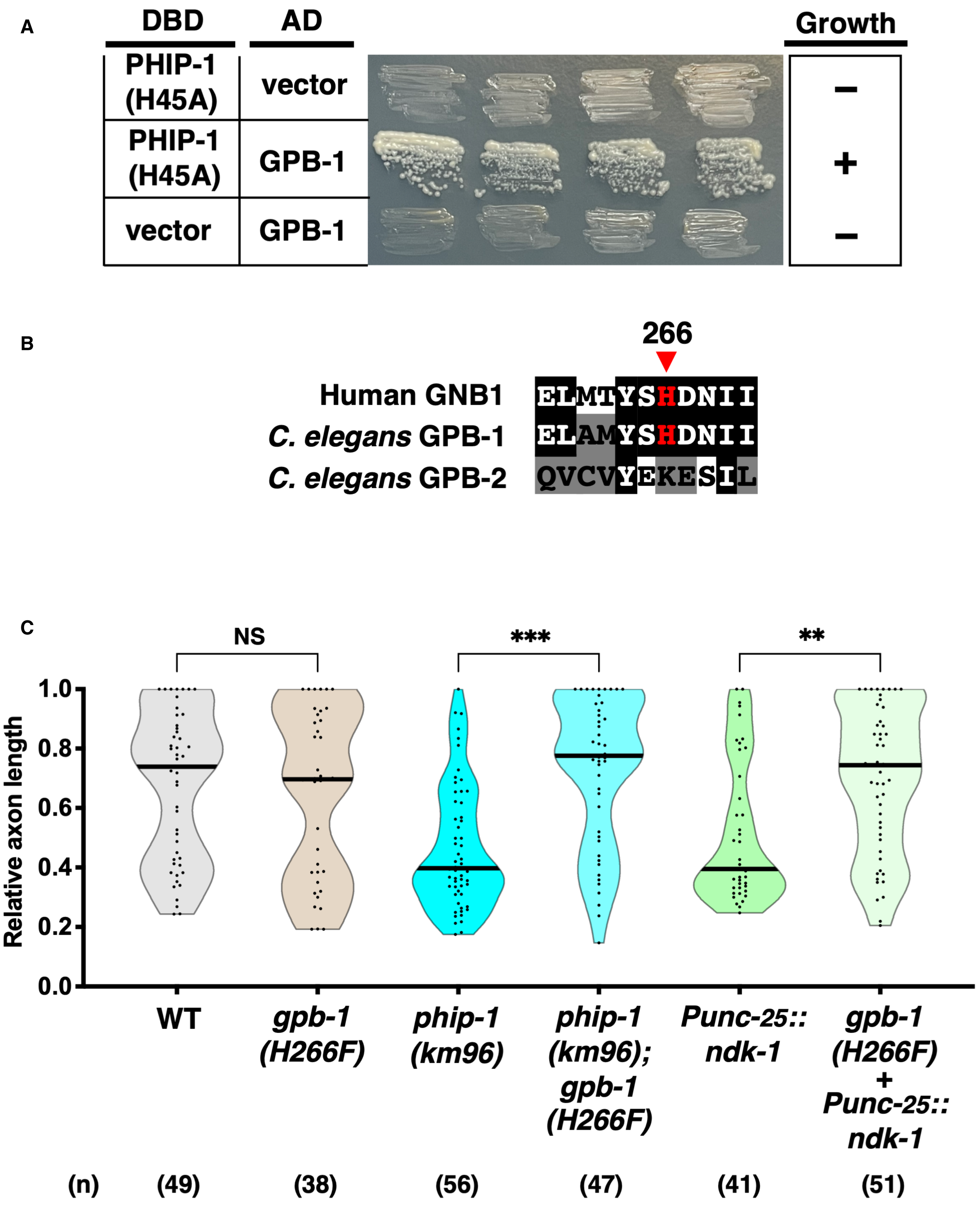

 © 2022 The Authors

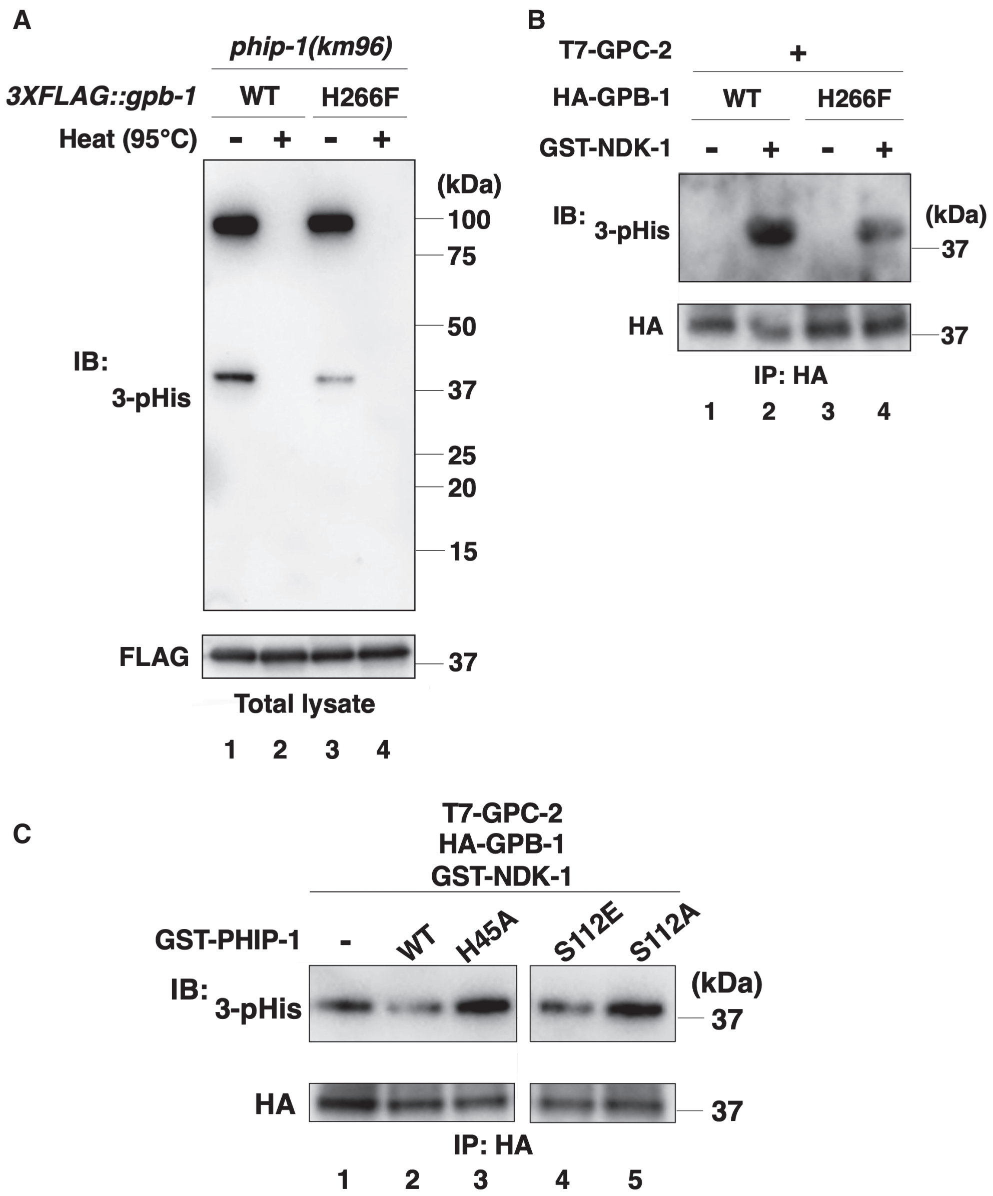

Figure 4. His-phosphorylation of GPB-1.

A His-phosphorylation of GPB-1 in animals. The *phip-1(km96)* mutant animals carrying the *3XFLAG::gpb-1* or *3XFLAG::gpb-1(H266F)* knock-in allele were lysed. The lysates were treated with or without heating (95°C) and immunoblotted (IB) with anti-3-pHis and anti-FLAG antibodies.

B NDK-1 phosphorylates GPB-1 *in vitro*. COS-7 cells were co-transfected with HA-GPB-1 or HA-GPB-1(H266F) and T7-GPC-2, and cell lysates were immunoprecipitated (IP) with anti-HA antibodies. Immunopurified GPB-1 was subjected to the *in vitro* kinase assay with recombinant GST-NDK-1. Phosphorylated GPB-1 was detected by immunoblotting (IB) with anti-3-pHis antibodies.

C PHIP-1 dephosphorylates GPB-1 *in vitro*. COS-7 cells were co-transfected with HA-GPB-1 and T7-GPC-2, and cell lysates were immunoprecipitated (IP) with anti-HA antibodies. The immunopurified HA-GPB-1 was first subjected to the *in vitro* kinase assay with recombinant GST-NDK-1. Phosphorylated GPB-1 was then equally aliquoted and subjected to the *in vitro* phosphatase assay with recombinant GST-PHIP-1 or its variants. Phosphorylated GPB-1 was detected by immunoblotting (IB) with anti-3-pHis antibodies.

phosphatase activity. Because NDK-1 is a housekeeping enzyme (Masoudi *et al*, 2013) and is presumed to be constitutively active, axon injury likely activates PHIP-1. We isolated a fragment (274–856 amino acids) of the serine/threonine kinase UNC-51, a *C. elegans* homolog of the mammalian autophagy-activating kinase ULK (Wang & Kundu, 2017), as a PHIP-1-binding protein (Fig 6A and B, Appendix Table S1). Because UNC-51 is a protein kinase, UNC-51 could regulate PHIP-1 function through phosphorylation. Therefore, we first examined whether UNC-51 phosphorylates PHIP-1 using mammalian cell culture. GFP-tagged UNC-51 and FLAG-tagged PHIP-1 were co-expressed in COS-7 cells, and cell lysates were subjected to phosphate-affinity (Phos-Tag) SDS–PAGE analysis, which can detect phosphorylated PHIP-1 by its slower migration in the gel. When wild-type GFP-UNC-51 was co-expressed with FLAG-PHIP-1

© 2022 The Authors

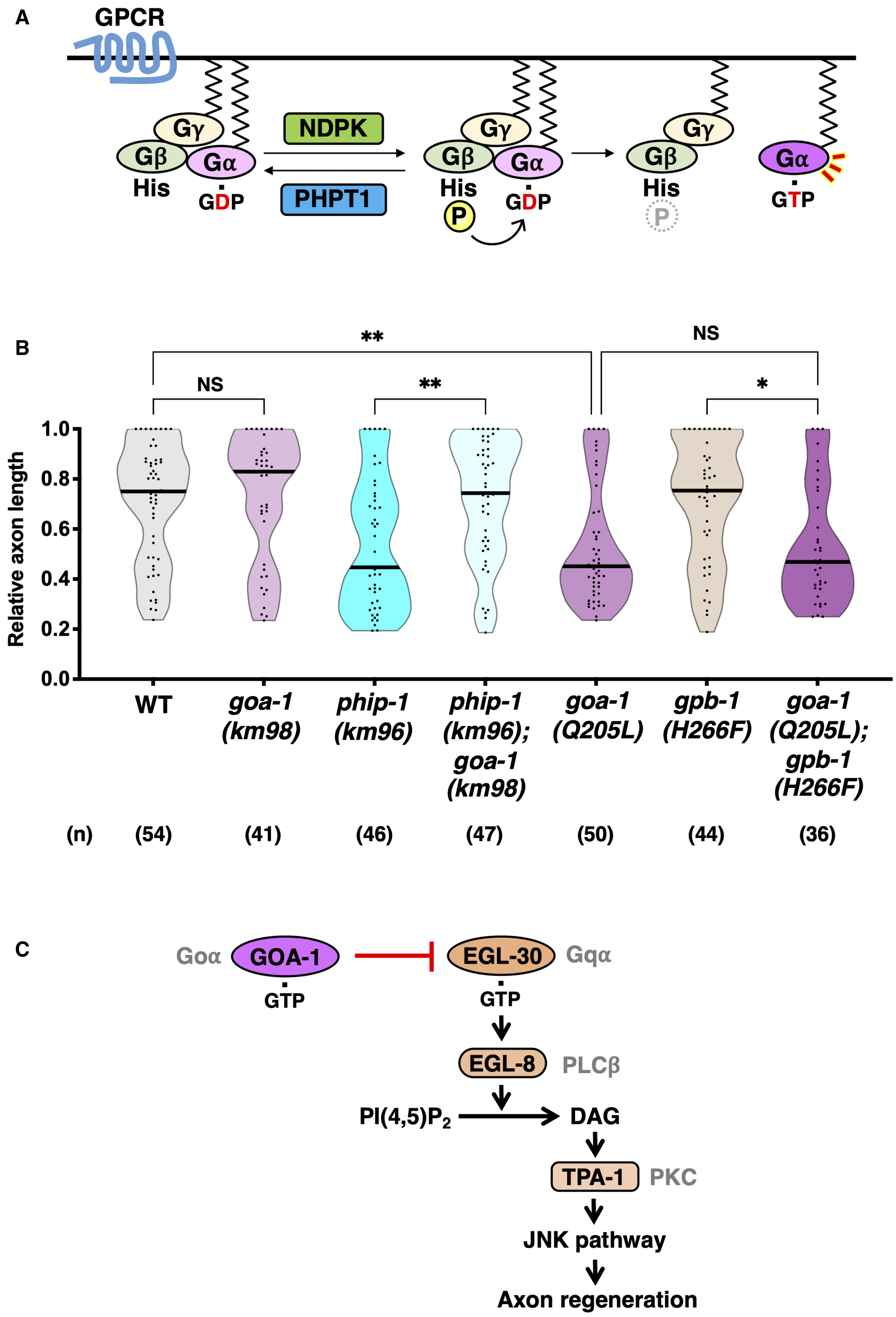

Figure 5.

 © 2022 The Authors

Figure 5. His-phosphorylation of GPB-1 inhibits axon regeneration by activating GOA-1 Goα.

A GPCR-independent Gα activation by His-phosphorylation of Gβ. NDPK phosphorylates Gβ, while PHPT1 counteracts this phosphorylation. When Gβ is His-phosphorylated, a high-energy pHis intermediate is transferred to GDP liganded to Gα, generating a GTP-bound form, which in turn activates G protein.

B Relative axon length 24 h after laser surgery at the young adult stage. The number (*n*) of axons examined from three biological replicates is shown. The black bar in each violin plot indicates the median. $^{*}P < 0.05$, $^{**}P < 0.01$, as determined by the Kruskal–Wallis test and Dunn's multiple comparison test. NS, not significant.

C The relationship between EGL-30 Gqα and GOA-1 Goα in axon regeneration. EGL-30 activates EGL-8 PLCβ, which in turn generates DAG from phosphatidylinositol bisphosphate [PI(4,5)P_2]. DAG activates TPA-1 PKC, resulting in the activation of the JNK pathway to promote axon regeneration. GTP-bound GOA-1 antagonizes the EGL-30 signaling cascade and inhibits axon regeneration. This inhibition is mediated by the phosphorylation of His-266 in GPB-1 Gβ, which leads to the activation of GOA-1 Goα signaling.

in COS-7 cells, PHIP-1 proteins appeared in the upper band (Fig 6C, lane 2). On the contrary, co-expression of the kinase-dead UNC-51 (ΔAIKAI), which lacks the catalytic lysine and four surrounding amino acids (Lai & Garriga, 2004), did not induce a mobility shift (Fig 6C, lane 3). These results indicate that the kinase activity of UNC-51 induces PHIP-1 phosphorylation. Then, we attempted to identify UNC-51 phosphorylation sites in PHIP-1, which contains seven serine and three threonine residues (Fig 6D). We created a series of FLAG-PHIP-1(S/T-A) mutant proteins, in which each Ser or Thr residue was replaced with alanine. We found that only the PHIP-1(S112A) variant did not appear in the upper band when co-expressed with GFP-UNC-51 (Fig 6C, lanes 4–13). These results indicate that UNC-51 phosphorylates PHIP-1 at Ser-112.

UNC-51 promotes axon regeneration by phosphorylating PHIP-1

To investigate the relationship between UNC-51 and PHIP-1 in axon regeneration, we first examined whether UNC-51 is required for axon regeneration in D-type motor neurons. However, the loss-of-function *unc-51* mutation was reported to severely affect the development of GABAergic D-type motor neurons (Ogura & Goshima, 2006; Appendix Fig S4A). Therefore, it is difficult to assess the effect of *unc-51(ks49)* on axon regeneration of D-type neurons. By contrast, the *unc-51* mutation has only a weak effect on axon elongation along the anterior–posterior axis of touch sensory posterior lateral microtubule (PLM) neurons (Appendix Fig S4B). Consistently, Chen *et al* (2011) recently identified *unc-51* among several genes that positively regulate axon regeneration in PLM neurons. We confirmed that *unc-51(ks49)* and *phip-1(km96)* mutants displayed impaired axon regeneration in PLM neurons (Fig 7A–C).

To clarify the physiological significance of UNC-51 phosphorylation of PHIP-1 Ser-112 on axon regeneration, we generated a phosphorylation-deficient *phip-1(S112A)* mutant at the endogenous *phip-1* locus using CRISPR–Cas9 mutagenesis (Appendix Fig S1A). We found that axon regeneration was significantly reduced in *phip-1(S112A)* mutants (Fig 7B and C). By contrast, introduction of the phosphomimetic *phip-1(S112E)* mutation into the *phip-1* locus (Appendix Fig S1A) partially suppressed the impaired regeneration in *unc-51(ks49)* mutants (Fig 7B and C). These results indicate that Ser-112 phosphorylation of PHIP-1 is involved in the UNC-51-mediated regeneration pathway. Next, we examined the genetic interaction between *unc-51* and *gpb-1* to determine whether UNC-51 promotes axon regeneration through GPB-1 pHis-266 dephosphorylation. Our result supports this possibility, because the nonphosphorylatable *gpb-1(H266F)* mutation partially suppressed the *unc-51(ks49)* phenotype (Fig 7B and C).

UNC-51 phosphorylation activates the catalytic activity of PHIP-1

We tested the effect of PHIP-1 Ser-112 phosphorylation on the catalytic activity of PHIP-1. We found that the phosphomimetic PHIP-1 (S112E) efficiently dephosphorylated pHis-GPB-1 *in vitro*, whereas phosphorylation-deficient PHIP-1(S112A) did not (Fig 4C, lane 4 and 5). These results indicate that Ser-112 phosphorylation of PHIP-1 activates its phosphatase activity. We further confirmed this possibility using recombinant GST-tagged bacterial histidine kinase CheA, which autophosphorylates itself on histidine (Klumpp *et al*, 2002; Wieland *et al*, 2010), as a substrate. We found that wild-type PHIP-1 weakly dephosphorylated pHis-CheA (Fig EV2, lane 1 and 3). By contrast, PHIP-1(S112E) showed stronger phosphatase activity than wild-type and PHIP-1(S112A) (Fig EV2, lane 3–5). These results suggest that Ser-112 phosphorylation of PHIP-1 is important for its catalytic activity.

Is the relationship between UNC-51 and PHIP-1 functionally conserved in mammals? To investigate this possibility, we performed an *in vitro* kinase assay using purified recombinant GST-tagged human ULK1. Immunopurified Myc-FLAG-tagged human PHPT1 from mammalian HEK293 cells was incubated with GST-ULK1 *in vitro*. We found that GST-ULK1 phosphorylates itself but not Myc-FLAG-PHPT1 (Appendix Fig S5A). Consistently, the Ser-112 residue in PHIP-1 corresponds to Ala-121 in PHPT1, but this site is not conserved (Appendix Fig S5B). However, the region surrounding Ser-112 in PHIP-1 is highly conserved with the corresponding region in PHPT1, and the Thr-119 residue is present in PHPT1 (Appendix Fig S5B). These findings suggest that an unknown kinase may activate PHPT1 activity by phosphorylating PHPT1 on Thr-119.

UNC-51 regulates axon regeneration via PHIP-1 and autophagy

The *unc-51(ks49)* mutant exhibited severe impairment in PLM axon regeneration, whereas the *phip-1(km96)* mutant showed a moderate reduction in axon regeneration (Fig 7B and C). These results suggest that UNC-51 regulates axon regeneration through other targets, in addition to PHIP-1. UNC-51 is also known to be involved in autophagy induction, and all autophagy mutants, including mutants of the gene that controls vesicle elongation (*lgg-2/LC3*), display impaired axon regeneration in PLM neurons (Ko *et al*, 2020). UNC-51/ULK kinase and LC3 are components of the primary autophagy machinery and are involved in the initiation of autophagosome biogenesis (Hurley & Young, 2017). Thus, although UNC-51/ULK and LGG-2/LC3 act in the same pathway in autophagy, the axon regeneration defect observed in *lgg-2(tm6544)* mutants was weaker than that in *unc-51(ks49)* mutants and comparable with that in *phip-1 (km96)* mutants (Fig EV3A and B). These results suggest that the

© 2022 The Authors

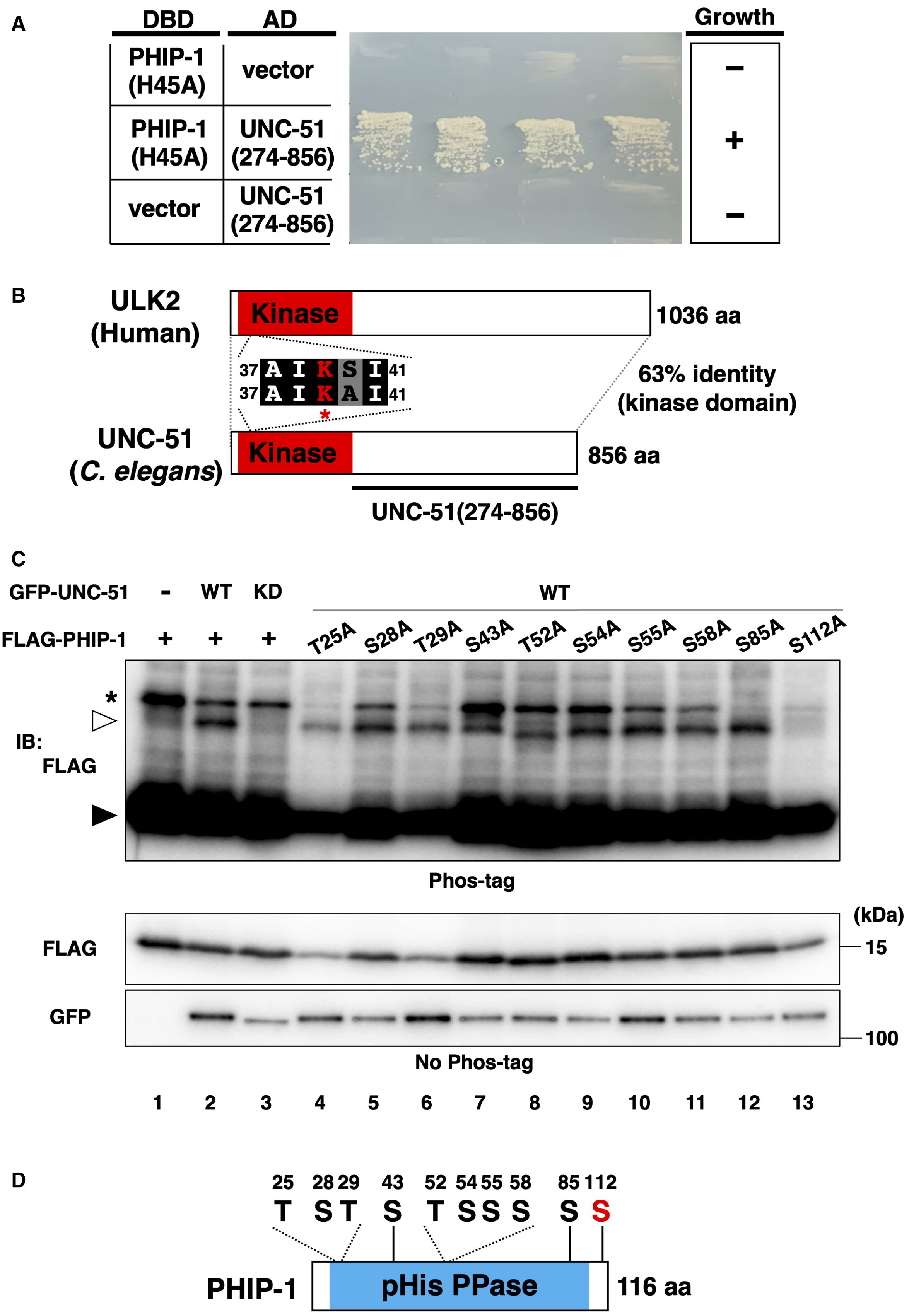

Figure 6.

© 2022 The Authors

Figure 6. UNC-51 phosphorylates PHIP-1 at Ser-112.

A PHIP-1 interaction with UNC-51 by yeast two-hybrid assay. The reporter strain PJ69-4A was co-transformed with expression vectors encoding GAL4 DBD-PHIP-1 (H45A) and GAL4 AD-UNC-51(274–856), as indicated. Yeast strains carrying the indicated plasmids were cultured on a selective plate lacking histidine and containing 5 mM 5-aminotriazole for 4 days.

B UNC-51 structure. Schematic diagrams of UNC-51 and human ULK2 are shown. The kinase domain is shown in red. The catalytic lysine and four flanking amino acids are shown. Identical and similar residues are highlighted with black and gray shading, respectively. The *unc-51(ks49)* mutation is a splice site mutation, which significantly reduces the *unc-51* mRNA level.

C UNC-51 phosphorylates PHIP-1 at Ser-112. COS-7 cells were co-transfected with Flag-PHIP-1 (WT or mutants) and GFP-UNC-51 [WT or ΔAIKAI (KD)], and cell lysates were analyzed using Phos-tag SDS–PAGE. Total lysates were immunoblotted (IB) with antibodies, as indicated. Filled and open arrowheads indicate unmodified and phosphorylated PHIP-1, respectively. Asterisk indicates nonspecific band.

D Schematic representation of the 10 Ser/Thr residues and domain structure in PHIP-1.

stronger impairment of axon regeneration in *unc-51* mutants is due to the inactivation of both PHIP-1 and the autophagy pathway (Fig EV3C). We found that the *phip-1(km96)*; *lgg-2(tm6544)* double mutant exhibited a greater reduction in regeneration than each mutation alone (Fig EV3A and B). Furthermore, although PHIP-1 acts downstream of UNC-51, the phosphomimetic *phip-1(S112E)* mutation partially suppressed *unc-51(ks49)* deficiency (Fig 7B and C). Thus, UNC-51 promotes axon regeneration through at least two independent pathways: one involving PHIP-1 and the other involving autophagy (Fig EV3C).

Discussion

His-phosphorylation has been used as a major mechanism to regulate signaling pathways in prokaryotes, some fungi, and plants (Attwood *et al*, 2007; Klumpp & Krieglstein, 2009). Recently, studies on His-phosphorylation in mammalian cells have revealed that His-phosphorylation of the ion channel Kca3.1 by NDPK is required for its function in T-cell activation (Srivastava *et al*, 2006), and PHIP-1 negatively regulates T-cell receptor signaling by dephosphorylating Kca3.1 (Srivastava *et al*, 2008). NDPK also phosphorylates and activates another cation ion channel TRPV5, facilitating intracellular Ca^{2+} reabsorption (Cai *et al*, 2014). In addition, His-phosphorylation of Gβ by NDPK is involved in Gsα-dependent cAMP synthesis in rat cardiomyocytes (Hippe *et al*, 2007). However, despite previous attempts to link His-phosphorylation, mammalian protein substrates, and cellular outcomes, the significance of His-phosphorylation for physiological functions in living animals remains largely unexplored. Taking advantage of the high conservation of His-kinases and pHis-phosphatases from worms to humans, we used *C. elegans* as an *in vivo* model animal. In this study, we discovered that His-phosphorylation is involved in axon regeneration, an evolutionarily conserved neuronal response, in which neurons regenerate damaged axons to restore their function. Our finding provides one of the best examples of how reversible His-phosphorylation regulates biological functions in living animals.

In vertebrate models, such as mouse and zebrafish, NDPK has been shown to participate in T-cell activation and cardiac contractility (Hippe *et al*, 2009; Di *et al*, 2010), suggesting that His-phosphorylation is involved in these processes. However, the causal relationship between His-phosphorylation and the observed phenotypes is complex because NDPK has a ubiquitous housekeeping function in nucleotide metabolism. This problem can be circumvented using pHis-phosphatases, PHPT1 and LHPP. PHPT1 is highly expressed in brain tissues and neurons and displays high enzymatic activity in rats (Klumpp *et al*, 2002), arguing its involvement in neuronal function. A recent study revealed that PHPT1 knockout mice developed hyperinsulinemic hypoglycemia in the neonatal period (Srivastava *et al*, 2018). LHPP seems to play a crucial role in CNS function and disease. An SNP in LHPP has been associated with major depressive disorder, alcohol dependence, and risky behavior (CONVERGE Consortium, 2015). Furthermore, a recent study demonstrated that LHPP overexpression suppresses tumorigenesis in a mouse model of hepatocellular carcinoma (Hindupur *et al*, 2018). In this study, we show that PHIP-1, a *C. elegans* ortholog of the mammalian PHPT1 protein, promotes axon regeneration in motor and sensory neurons. Thus, pHis-phosphatase can be used as an entry point to identify the physiological roles of His-phosphorylation in living animals.

How does PHIP-1 promote axon regeneration? We discovered that the His-kinase NDK-1 inhibits axon regeneration by phosphorylating GPB-1 Gβ at the His-266 residue. Our genetic and biochemical data indicate that PHIP-1 counteracts GPB-1 phosphorylation, thereby inducing regeneration. Mammalian NDPK and PHPT1 phosphorylate and dephosphorylate His-266 of GNB1 Gβ, respectively (Hippe *et al*, 2003), indicating that the Gβ His-phosphorylation–dephosphorylation system is conserved between *C. elegans* and mammals. His-phosphorylation of Gβ has been shown to induce Gα activation in a GPCR-independent manner (Cuello *et al*, 2003; Hippe *et al*, 2003, 2007); however, whether this is physiologically important is unclear. Here, we demonstrate that His-phosphorylation of GPB-1 inhibits axon regeneration through the activation of the Goα GOA-1, which antagonizes the EGL-30 Gqα signaling pathway (Pastuhov *et al*, 2012). This reveals a genetic link between Gβ His-phosphorylation and Gα activation. The activation of Gα by the generation of the GTP-bound form through the His-phosphorylated Gβ intermediate regulates receptor-independent activation of heterotrimeric G proteins. Therefore, it is reasonable to assume that His-phosphorylation of Gβ has the potential to regulate a broad spectrum of cellular functions.

In this study, we report for the first time that the signaling event required for axon regeneration involves the activation of pHis-phosphatase. An important question is how this activation is regulated in animals. We found that UNC-51 kinase promotes axon regeneration by activating pHis-phosphatase activity through the phosphorylation of PHIP-1 Ser-112. Under normal conditions, PHIP-1 is inactive. Axon injury activates UNC-51, which in turn activates PHIP-1 through phosphorylation. A recent study has revealed that RPM-1, an E3 ubiquitin ligase, negatively regulates UNC-51, affecting axon termination, synapse maintenance, and behavioral habituation (Crawley *et al*, 2019). Thus, RPM-1 restricts UNC-51 and exerts its effects broadly across the nervous system. Interestingly, RPM-1 is known to negatively regulate axon regeneration (Yan

© 2022 The Authors

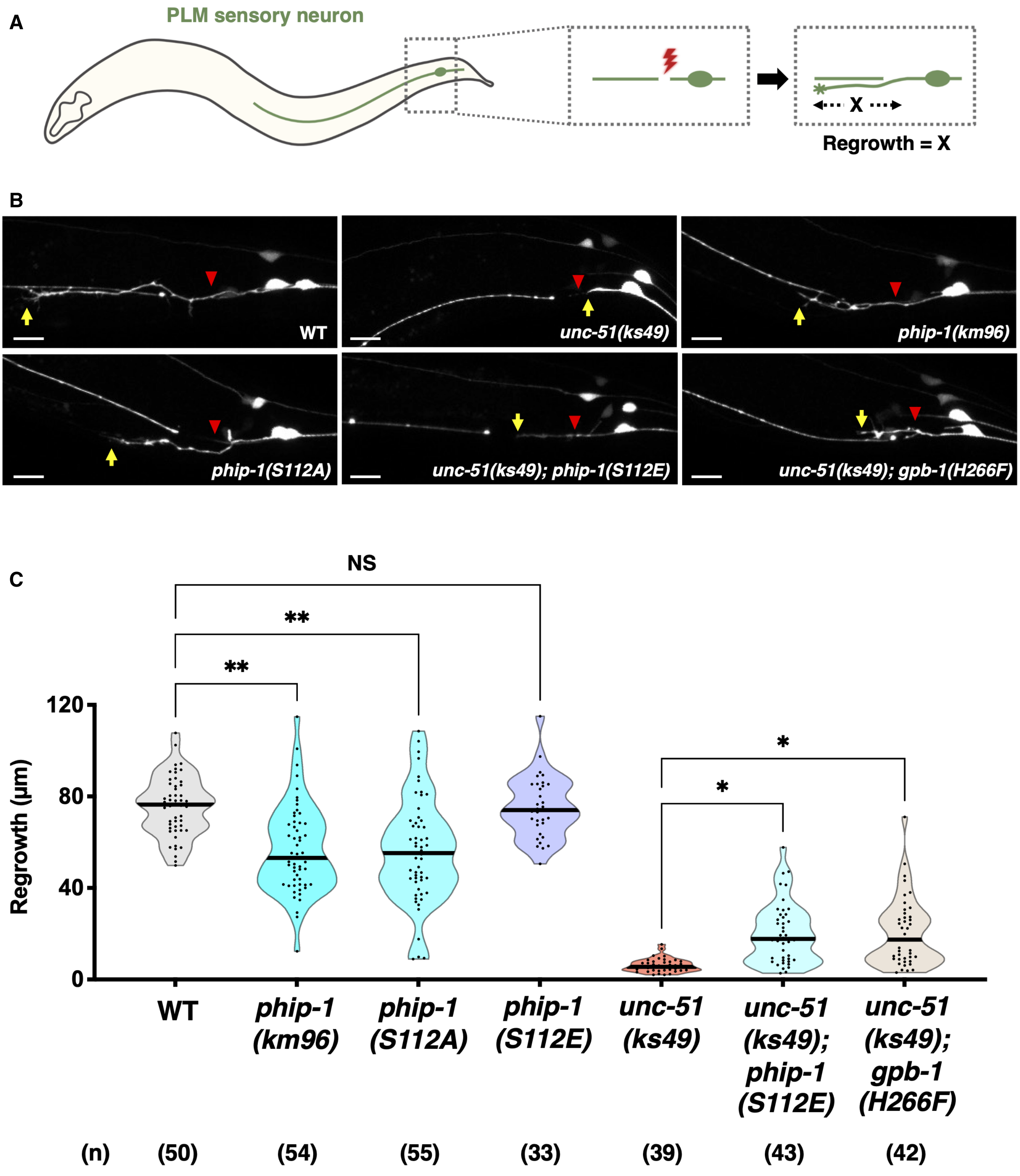

Figure 7. UNC-51 promotes axon regeneration by phosphorylating PHIP-1.

A Scheme for axotomy of PLM sensory neurons in *Caenorhabditis elegans*.

B Representative PLM sensory neurons in indicated genotypes 24 h after laser surgery. Red arrowheads indicate cut sites. Yellow arrows indicate the tip of axotomized axons. Scale bar, 10 μm.

C Length of PLM regrowth 24 h after laser surgery. The number (*n*) of axons examined from three biological replicates is indicated. The black bar in each violin plot indicates the median. $^*P < 0.05$, $^{**}P < 0.01$, as determined by the Kruskal–Wallis test and Dunn's multiple comparison test. NS, not significant.

 © 2022 The Authors

et al, 2009). Based on these findings, we propose that axon injury induces the stabilization of UNC-51 and activates its kinase activity. Activated UNC-51 phosphorylates PHIP-1 Ser-112, thereby activating its pHis-phosphatase activity. Therefore, the activation of PHIP-1 by UNC-51 phosphorylation appears to be specific for axon regeneration. If so, PHIP-1 activity would be activated under conditions that would activate a kinase capable of phosphorylating PHIP-1 Ser-112. Is such a system at work in mammals? Similar to the *C. elegans* *phip-1* deletion mutant, siRNA depletion of the PHPT1 pHis-phosphatase in HeLa cells did not result in a gross increase in the number of pHis peptides (Hardman *et al*, 2019), suggesting that PHPT1 is inactive under normal conditions. The region surrounding PHIP-1 Ser-112 is also highly conserved in mammalian PHPT1, where Thr-119 is located near Ser-112 site of PHIP-1. Considering these results, it is possible that PHPT1 is activated through Thr-119 phosphorylation.

Materials and Methods

C. elegans strains

The *C. elegans* strains used in this study are listed in Appendix Table S2. All strains were maintained on nematode growth medium plates and fed with bacteria of the OP50 strain using the standard method (Brenner, 1974).

Plasmids

Punc-25::phip-1 and *Punc-25::ndk-1* were generated by inserting the *phip-1* and *ndk-1* cDNAs isolated from the cDNA library into the pSC325 vector, respectively. *Punc-25::phip-1(H45A)* and *Punc-25::ndk-1(H118N)* were generated by oligonucleotide-directed inverse PCR using *Punc-25::phip-1* and *Punc-25::ndk-1* as templates, respectively, and the mutations were verified using DNA sequencing. The HA-GPB-1, T7-GPC-2, FLAG-PHIP-1, GST-PHIP-1, GST-NDK-1, and GFP-UNC-51 plasmids were generated by inserting the *gpb-1*, *gpc-2*, *phip-1*, *ndk-1*, and *unc-51* cDNAs isolated from the cDNA library into the pCMV-HA, pCMV-T7, pCMV-FLAG, pGEX-6P-1, and pEGFP-C1 vectors, respectively. The GST-CheA plasmid was generated by inserting the bacterial CheA DNA isolated from OP50 *E. coli* into the pGEX-6P-1 vector. The Myc-FLAG-PHPT1 plasmid was purchased from OriGene (RC205124). The FLAG-PHIP-1(S112A), FLAG-PHIP-1(S112E), GST-PHIP-1(S112A), GST-PHIP-1(S112E), GST-PHIP-1(H45A), HA-GPB-1(H266F), and GFP-UNC-51(ΔAIKAI) plasmids were generated by oligonucleotide-directed inverse PCR, and the mutations were verified using DNA sequencing. The pDBD-PHIP-1(H45A) plasmid was generated by inserting the *phip-1(H45A)* cDNA into pGBDU-C. The pAD-GPB-1 and pAD-UNC-51(274–856) plasmids were generated by inserting *gpb-1* and *unc-51* cDNAs into pACTII, respectively. The *Pmyo-2::dsred-monomer* has been described (Li *et al*, 2012).

Genome editing using CRISPR–Cas9

The genome editing by CRISPR–Cas9 was performed, as previously described (Dokshin *et al*, 2018). To generate the *phip-1(km96)*, *lhpp-1(km97)*, *goa-1(km98)*, *gpb-1(H266F)*, *phip-1(S112A)*, *phip-1(S112E)*, and *3XFLAG::gpb-1* alleles, CRISPR RNA sequences targeting the mutation or insertion region were designed and synthesized from Integrated DNA Technologies (IDT). Single-stranded donor template DNA (ssDNA) was also designed and synthesized for generating the *gpb-1(H226F)*, *phip-1(S112A)*, *phip-1(S112E)*, and *3XFLAG::gpb-1* alleles. Furthermore, CRISPR RNA, tracer RNA (IDT), and Cas9 nuclease (IDT) were co-injected in the gonads with the donor DNA when necessary, and F1 animals carrying an injection marker pRF4(*rol-6d*) were transferred onto a new dish and used for single-worm PCR, followed by DNA sequencing to detect mutations and insertions. The oligonucleotides used to create and detect each mutant are listed in Appendix Table S3.

Transgenic animals

Transgenic animals were obtained using the standard *C. elegans* microinjection method (Mello *et al*, 1991). *Pmyo-2::dsred-monomer*, *Punc-25::phip-1*, *Punc-25::phip-1(H45A)*, *Punc-25::ndk-1*, and *Punc-25::ndk-1(H118N)* plasmids were used in *kmEx1461* [*Punc-25::phip-1* (5 ng/μl) + *Pmyo-2::dsred-monomer* (5 ng/μl)], *kmEx1462* [*Punc-25::phip-1(H45A)* (5 ng/μl) + *Pmyo-2::dsred-monomer* (5 ng/μl)], *kmEx1463* [*Punc-25::ndk-1* (50 ng/μl) + *Pmyo-2::dsred-monomer* (5 ng/μl)], and *kmEx1464* [*Punc-25::ndk-1(H118N)* (50 ng/μl) + *Pmyo-2::dsred-monomer* (5 ng/μl)], respectively. The *juIs76* and *muIs32* integrated arrays were previously described (Huang *et al*, 2002; Ch'ng *et al*, 2003).

Microscopy

Standard fluorescent images of transgenic animals were observed under an ×100 objective of a Nikon ECLIPSE E800 fluorescent microscope and captured using a Zyla CCD camera. Confocal fluorescent images were taken on a Zeiss LSM-800 confocal laser-scanning microscope with ×40 or ×63 objective.

Axotomy

Axotomy and microscopy were performed as previously described (Li *et al*, 2012). GABAergic D-type motor neuron commissures (VD9, DD5, and VD10) and touch sensory PLM neurons were labeled by *juIs76* and by *muIs32*, respectively. D-type motor neuron commissures were targeted at the dorsoventral midline, and PLM neurons were targeted at 15–25 μm from soma. The *unc-51(ks49)* mutant animals, with short PLM axons, were excluded from the assay. Animals were subjected to axotomy at the young adult stage. The young adult stage was defined as a state, in which the vulva is well developed and no eggs have formed yet. Animals were recovered to NGM plates seeded with OP50 *E. coli* and analyzed for axon regeneration 24 h after axotomy.

Analysis of axon regeneration

To analyze D-type motor neuron regeneration, the relative length of axotomized axons was measured using ImageJ and plotted with GraphPad Prism 9. Relative axon length was determined by the distance from the ventral nerve cord to the injured axon tip normalized

© 2022 The Authors

by the distance from the ventral nerve cord to the dorsal nerve cord. To analyze the regrowth of D-type motor neurons and PLM neurons, the lengths of the regenerating axons were measured using the segmented line tool of ImageJ. Measurements were made from the site of injury to the tip of the longest branch of the regenerating axon. Data were plotted using GraphPad Prism 9.

Yeast two-hybrid screen and analysis

Yeast two-hybrid screen using the GAL4 DBD-PHIP-1(H45A) plasmid was performed as previously described (Sakamoto *et al*, 2005). For yeast two-hybrid analysis, GAL4 AD-GPB-1, GAL4 AD-UNC-51 (274-856), or pACTII plasmids were co-transformed with either GAL4 DBD-PHIP-1(H45A) or the pGBDU-C vector into the *Saccharomyces cerevisiae* reporter strain PJ69-4A (*MATa trp1-901 ura3-52 leu2-3,112 his3-200 gal4Δ gal80Δ met2::GAL7-lacZ LYS2::GAL1-HIS3 ade2::GAL2-ADE2*), and yeasts were allowed to grow on SC-Ura-Leu plates. Then, transformants cultured on these plates were streaked out onto SC-Ura-Leu-His plates with 5 mM 5-aminotriazole and incubated at 30°C for 4 days.

Detection of GPB-1 His-phosphorylation

To extract 3XFLAG::GPB-1 from worms, the worms were collected from NGM plates with M9 medium, suspended in cold RIPA buffer [50 mM Tris–HCl (pH 8.0), 150 mM NaCl, 1% NP-40, 0.5% sodium deoxycholate, 0.1% SDS, 5 mM PMSF, phosphatase inhibitor cocktail 2 and 3 (Sigma-Aldrich), and protease inhibitor cocktail (Sigma-Aldrich)] and sonicated using Bioraptor UCW-201 (Cosmo Bio) at 4°C. After sonication, the samples were centrifuged at 15,000 × *g* for 15 min at 4°C. The supernatant was mixed with SDS sample buffer (AE-1430; ATTO) and used as total lysate for western blotting. Western blotting for pHis was performed as described (Kalagiri *et al*, 2020). The primary antibodies used were as follows: mouse monoclonal anti-FLAG (M2; Sigma-Aldrich), rabbit monoclonal anti-1-pHis (clone SC50-3; Sigma-Aldrich; Fuhs *et al*, 2015), and rabbit monoclonal anti-3-pHis (clone SC39-6; Sigma-Aldrich; Fuhs *et al*, 2015).

In vitro kinase and phosphatase assays

The GST-ULK1 recombinant protein was purchased from SignalChem (U01-11G). GST-NDK-1, GST-PHIP-1(WT/H45A/S112A/S112E), and GST-CheA recombinant proteins were expressed in SoluBL21 Competent *E. coli* (Genlantis) and purified using glutathione-Sepharose 4B (GE Healthcare), following the manufacturer's guidelines. HA-GPB-1(WT/H266F) was immunopurified with the anti-HA (16B12; BioLegend) antibody from COS-7 cells expressing HA-GPB-1(WT/H266F) and T7-GPC-2 plasmids. Myc-FLAG-PHPT1 protein was immunopurified using the anti-FLAG (M2; Sigma-Aldrich) antibody from HEK293 cells. All kinase reactions were performed in a final volume of 20 µl buffer consisting of 50 mM HEPES (pH 7.4), 5 mM $MgCl_2$, 5 mM $MnCl_2$, 0.5 mM DTT, and 100 µM ATP. When phosphorylation was analyzed by autoradiography, 5 µCi of [γ-^{32}P]ATP was also added. Kinase reaction samples were incubated for 20 min (PHPT1 phosphorylation), 60 min (GPB-1 phosphorylation), or 180 min (CheA autophosphorylation) at 30°C. For the *in vitro* phosphatase assays, wild-type or mutant GST-PHPT-1 was added to the kinase reaction samples after the kinase reaction was complete, and the samples were incubated for 30 min at 30°C. Kinase or phosphatase reactions were terminated by the addition of Laemmli sample buffer without boiling, except for the PHPT1 phosphorylation sample. The samples were resolved by SDS–PAGE and analyzed by autoradiography or subjected to immunoblotting. The primary antibodies used were as follows: mouse monoclonal anti-HA (16B12; BioLegend), rabbit monoclonal anti-1-pHis (clone SC50-3; Sigma-Aldrich; Fuhs *et al*, 2015), and rabbit monoclonal anti-3-pHis (clone SC39-6; Sigma-Aldrich; Fuhs *et al*, 2015).

Phos-tag assay

Transfected COS-7 cells were lysed in RIPA buffer [50 mM Tris–HCl (pH 7.4), 0.15 M NaCl, 0.25% deoxycholic acid, 1% NP-40, 1 mM EDTA, 1 mM dithiothreitol, 1 mM phenylmethylsulfonyl fluoride, phosphatase inhibitor cocktail 2 and 3 (Sigma-Aldrich), and protease inhibitor cocktail (Sigma-Aldrich)], followed by centrifugation at 15,000 × *g* for 12 min. For SDS–PAGE, 15% Phos-tag precast gel (SuperSep Phos-tag, Wako) was used. After electrophoresis, Phos-tag acrylamide gels were washed three times and stirred gently in transfer buffer (Fast buffer, ATTO) containing 0.01% SDS and 10 mM EDTA for 10 min. Then, it was incubated in the transfer buffer containing 0.01% SDS without EDTA for 10 min according to the manufacturer's protocol. Proteins were transferred to polyvinylidene difluoride (PVDF) membranes and immunoblotted with the anti-Flag (M2; Sigma-Aldrich) or anti-GFP (mouse JL-8; Clontech) antibody. The bound antibodies were visualized with horseradish peroxidase (HRP)-conjugated antibody against mouse IgG using the HRP chemiluminescent substrate reagent kit (Novex ECL; Invitrogen).

Aldicarb assay

Aldicarb-induced paralysis assay was performed as previously described (Mahoney *et al*, 2006). One-day-young adult worms were incubated on NGM plates containing 1 mM aldicarb with a small spot of OP50 *E. coli* solution, and the fraction of paralyzed worms was counted every 30 min for 4 h. Animals with no response to touch stimulation and no pharyngeal pumping were considered paralyzed and removed from the plate. Assays were performed blindly and in triplicate, and significance was determined by a log-rank test using Prism 9.

Experimental design and statistical analyses

All experiments were not randomized, and the investigators were not blinded to the group allocation during the experiments and outcome assessments except for the aldicarb assay. No statistical method was used to predetermine sample size. Data visualization was performed using GraphPad Prism 9. In addition, statistical analyses were performed using GraphPad Prism 9, R (ver. 4.0.1) and R studio (ver. 1.3.959). Two-tailed *P*-values were calculated by the Mann–Whitney test, Kruskal–Wallis test, and Dunn's multiple comparison test. The log-rank test was used to compare the overall curve.

 © 2022 The Authors

Data availability

No primary datasets were generated or deposited in public databases.

Expanded View for this article is available online.

Acknowledgements

We thank the *Caenorhabditis* Genetic Center (CGC), National Bio-Resource Project and *C. elegans* Knockout Consortium for materials. Some strains were provided by the CGC, which is funded by NIH Office of Research Infrastructure Programs (P40 OD10440). This work was supported by grants from the Ministry of Education, Culture and Science of Japan (to KM), Japan Society for the Promotion of Science, under Grant Number 22H04643 (to NH) and the Project for Elucidating and Controlling Mechanisms of Aging and Longevity from Japan Agency for Medical Research and Development, AMED, under Grant Number JP21gm5010001 (to NH). YS was supported by a Japan Society for the Promotion of Science Research Fellowship, under Grant Number 21J15589.

Author contributions

Yoshiki Sakai: Conceptualization; funding acquisition; validation; investigation; visualization; methodology; writing – original draft. **Hiroshi Hanafusa:** Investigation. **Naoki Hisamoto:** Funding acquisition; project administration. **Kunihiro Matsumoto:** Conceptualization; supervision; funding acquisition; project administration; writing – review and editing.

Disclosure and competing interests statement

The authors declare that they have no conflict of interest.

References

Afshar K, Willard FS, Colombno K, Johnston CA, McCudden CR, Siderovski DP, Gönczy P (2004) RIC-8 is required for GPR-1/2-dependent Gα function during asymmetric division of *C. elegans* embryos. *Cell* 119: 219–230

Attwood PV, Piggott MJ, Zu XL, Besant PG (2007) Focus on phosphohistidine. *Amino Acids* 32: 145–156

Brenner S (1974) The genetics of *Caenorhabditis elegans*. *Genetics* 77: 71–94

Busam RD, Thorsell A-G, Flores A, Hammarström M, Persson C, Hallberg BM (2006) First structure of a eukaryotic phosphohistidine phosphatase. *J Biol Chem* 281: 33830–33834

Cai X, Srivastava S, Surindran S, Li Z, Skolnik EY (2014) Regulation of the epithelial Ca^{2+} channel TRPV5 by reversible histidine phosphorylation mediated by NDPK-B and PHPT1. *Mol Biol Cell* 25: 1244–1250

Chen L, Wang Z, Ghosh-Roy A, Hubert T, Yan D, O'Rourke S, Bowerman B, Jin Y, Chisholm AD (2011) Axon regeneration pathways identified by systematic genetic screening in *C. elegans*. *Neuron* 71: 1043–1057

Ch'ng Q, Williams L, Lie YS, Sym M, Whangbo J, Kenyon C (2003) Identification of genes that regulate a left-right asymmetric neuronal migration in *Caenorhabditis elegans*. *Genetics* 164: 1355–1367

CONVERGE Consortium (2015) Sparse whole-genome sequencing identifies two loci for major depressive disorder. *Nature* 523: 588–591

Crawley O, Opperman KJ, Desbois M, Adrados I, Borgen MA, Giles AC, Duckett DR, Grill B (2019) Autophagy is inhibited by ubiquitin ligase activity in the nervous system. *Nat Commun* 10: 5017

Cuello F, Schulze R, Heemeyer F, Meyer HE, Lutz S, Jakobs KH, Nicroomand F, Wieland T (2003) Activation of heterotrimeric G proteins by a high energy phosphate transfer via nucleoside diphosphate kinase (NDPK) B and Gβ subunits. *J Biol Chem* 278: 7220–7226

Di L, Srivastava S, Zhdanova O, Sun Y, Li Z, Skolnik EY (2010) Nucleoside diphosphate kinase B knock-out mice have impaired activation of the K^+ channel Kca3.1, resulting in defective T cell activation. *J Biol Chem* 285: 38765–38771

Dokshin GA, Ghanta KS, Piscopo KM, Mello CC (2018) Robust genome editing with short single-stranded and long, partially single-stranded DNA donors in *Caenorhabditis elegans*. *Genetics* 210: 781–787

Ek P, Petterson G, Ek B, Gong F, Li J-P, Zetterqvist O (2002) Identification and characterization of a mammalian 14-kDa phosphohistidine phosphatase: a mammalian 14-kDa phosphohistidine phosphatase. *Eur J Biochem* 269: 5016–5023

Fuhs SR, Hunter T (2017) pHisphorylation: the emergence of histidine phosphorylation as a reversible regulatory modification. *Curr Opin Cell Biol* 45: 8–16

Fuhs SR, Meisenhelder J, Aslanian A, Ma L, Zagorska A, Stankova M, Binnie A, Al-Obeidi F, Mauger J, LemKe Gal-Obeidi F *et al* (2015) Monoclonal 1- and 3-phosphohistidine antibodies: new tools to study histidine phosphorylation. *Cell* 162: 198–210

Gotta M, Ahringer J (2001) Distinct roles for Gβ and Gβγ in regulating spindle position and orientation in *Caenorhabditis elegans* embryos. *Nat Cell Biol* 3: 297–300

Hardman G, Perkins S, Brownridge PJ, Clarke CJ, Byrne DP, Campbell AE, Kalyuzhnyy A, Myall A, Eyers PA, Jones AR *et al* (2019) Strong anion exchange-mediated phosphoproteomics reveals extensive human non-canonical phosphorylation. *EMBO J* 38: e100847

Hartsough MT, Morrison DK, Salerno M, Palmieri D, Ouatas T, Mair M, Patrick J, Steeg PS (2002) Nm23-H1 metastasis suppressor phosphorylation of kinase suppressor of Ras via a histidine protein kinase pathway. *J Biol Chem* 277: 32389–32399

Hess JF, Bourret RB, Simon MI (1988) Histidine phosphorylation and phosphoryl group transfer in bacterial chemotaxis. *Nature* 336: 139–143

Hess HA, Roper JC, Grill SW, Koelle MR (2004) RGS-7 completes a receptor-independent heterotrimeric G protein cycle to asymmetrically regulate mitotic spindle positioning in *C. elegans*. *Cell* 119: 209–218

Hindupur SK, Colombi M, Fuhs SR, Matter MS, Guri Y, Adam K, Cornu M, Piscuoglio S, Ng CKY, Betz C *et al* (2018) The protein histidine phosphatase LHPP is a tumour suppressor. *Nature* 555: 678–682

Hippe H-J, Lutz S, Cuello F, Knorr K, Vogt A, Jakobs KH, Wieland T, Niroomand F (2003) Activation of heterotrimeric G proteins by a high energy phosphate transfer via nucleoside diphosphate kinase (NDPK) B and Gβ subunits. *J Biol Chem* 278: 7227–7233

Hippe H-J, Luedde M, Lutz S, Koehler H, Eschenhagen T, Frey N, Katus H, Wieland T, Niroomand F (2007) Regulation of cardiac cAMP synthesis and contractility by nucleoside diphosphate kinase B/G protein βγ dimer complexes. *Circ Res* 100: 1191–1199

Hippe H-J, Wolf NM, Abu-Taha I, Mehringer R, Just S, Lutz S, Niroomand F, Postel EH, Katus HA, Rottbauer W *et al* (2009) The interaction of nucleoside diphosphate kinase B with Gβγ dimers controls heterotrimeric G protein function. *Proc Natl Acad Sci USA* 106: 16269–16274

Huang X, Cheng HJ, Tessier-Lavigne M, Jin Y (2002) MAX-1, a novel PH/$MyTH_4$/FERM domain cytoplasmic protein implicated in netrin-mediated axon repulsion. *Neuron* 34: 563–576

Hurley JH, Young LN (2017) Mechanisms of autophagy initiation. *Annu Rev Biochem* 86: 225–244

© 2022 The Authors

Jansen G, Weinkove D, Plasterk RH (2002) The G protein γ-subunit *gpc-1* of the nematode *C. elegans* is involved in taste adaptation. *EMBO J* 21: 986 – 994

Kalagiri R, Adam K, Hunter T (2020) Empirical evidence of cellular histidine phosphorylation by immunoblotting using pHis mAbs. *Methods Mol Biol* 2077: 181 – 191

Klumpp S, Krieglstein J (2009) Reversible phosphorylation of histidine residues in proteins from vertebrates. *Sci Signal* 2: pe13

Klumpp S, Hermesmeier J, Selke D, Baumeister R, Kellner R, Krieglstein J (2002) Protein histidine phosphatase: a novel enzyme with potency for neuronal signaling. *J Cereb Blood Flow Metab* 22: 1420 – 1424

Ko S-H, Apple EC, Liu Z, Chen L (2020) Age-dependent autophagy induction after injury promotes axon regeneration by limiting NOTCH. *Autophagy* 16: 2052 – 2068

Lai T, Garriga G (2004) The conserved kinase UNC-51 acts with VAB-8 and UNC-14 to regulate axon outgrowth in *C. elegans*. *Development* 131: 5991 – 6000

Lecroisey A, Lascu I, Bominaar A, Veron M, Delepierre M (1995) Phosphorylation mechanism of nucleoside diphosphate kinase: ^{31}P-nuclear magnetic resonance studies. *Biochemistry* 34: 12445 – 12450

Li C, Hisamoto N, Nix P, Kanao S, Mizuno T, Bastiani M, Matsumoto K (2012) The growth factor SVH-1 regulates axon regeneration in *C. elegans* via the JNK MAPK cascade. *Nat Neurosci* 15: 551 – 557

Lu Z, Hunter T (2018) Metabolic kinases moonlighting as protein kinases. *Trends Biochem Sci* 43: 301 – 310

Mahoney TR, Luo S, Nonet ML (2006) Analysis of synaptic transmission in *Caenorhabditis elegans* using an aldicarb-sensitivity assay. *Nat Protoc* 1: 1772 – 1777

Masoudi N, Fancsalszky L, Pourkarimi E, Vellai T, Alexa A, Reményi A, Gartner A, Mehta A, Takács-Vellai K (2013) The NM23-H1/H2 homolog NDK-1 is required for full activation of Ras signaling in *C. elegans*. *Development* 140: 3486 – 3495

Mello CC, Kramer JM, Stinchcombm D, Ambros V (1991) Efficient gene transfer in *C. elegans*: extrachromosomal maintenance and integration of transforming sequences. *EMBO J* 10: 3959 – 3970

Ogura K, Goshima Y (2006) The autophagy-related kinase UNC-51 and its binding partner UNC-14 regulate the subcellular localization of the netrin receptor UNC-5 in *Caenorhabditis elegans*. *Development* 133: 3441 – 3450

Oldham WM, Hamm HE (2008) Heterotrimeric G protein activation by G-protein-coupled receptors. *Nat Rev Mol Cell Biol* 9: 60 – 71

Panda S, Srivastava S, Li Z, Vaeth M, Fuhs SR, Hunter T, Skolnik EY (2016) Identification of PGAM5 as a mammalian protein histidine phosphatase that plays a central role to negatively regulate $CD4^+$ T cells. *Mol Cell* 63: 457 – 469

Pastuhov SI, Fujiki K, Nix P, Kanao S, Bastiani M, Matsumoto K, Hisamoto N (2012) Endocannabinoid–Goα signalling inhibits axon regeneration in *Caenorhabditis elegans* by antagonizing Gqα–PKC–JNK signalling. *Nat Commun* 3: 1136

Robatzek M, Niacaris T, Steger K, Avery L, Thomas JH (2001) *eat-11* encodes GPB-2, a Gβ5 ortholog that interacts with Goα and Gqα to regulate *C. elegans* behavior. *Curr Biol* 11: 288 – 293

Sakamoto R, Byrd DT, Brown HM, Hisamoto N, Matsumoto K, Jin Y (2005) The *Caenorhabditis elegans* UNC-14 RUN domain protein binds to the kinesin-1 and UNC-16 complex and regulates synaptic vesicle localization. *Mol Biol Cell* 16: 483 – 496

Srivastava S, Li Z, Ko K, Choudury P, Albaqumi M, Johnson AK, Yan Y, Backer JM, Unutmaz D, Coetzee WA *et al* (2006) Histidine phosphorylation of the potassium channel KCa3.1 by nucleoside diphosphate kinase B is required for activation of KCa3.1 and CD4 T cells. *Mol Cell* 24: 665 – 675

Srivastava S, Zhdanova O, Skolnik EY (2008) Protein histidine phosphatase 1 negatively regulates CD4 T cells by inhibiting the K^+ channel KCa3.1. *Proc Natl Acad Sci USA* 105: 14442 – 14446

Srivastava S, Panda S, Li Z, Fuhs SR, Hunter T, Thiele DJ, Hubbrd SR, Skolnik EY (2016) Histidine phosphorylation relieves copper inhibition in the mammalian potassium channel KCa3.1. *Elife* 5: e16093

Srivastava S, Li Z, Soomro I, Sun Y, Wang J, Bao L, Coetzee WA, Stanley CA, Li C, Skolnik EY (2018) Regulation of K_{ATP} channel trafficking in pancreatic β-cells by protein histidine phosphorylation. *Diabetes* 67: 849 – 860

Swanson RV, Alex LA, Simon MI (1994) Histidine and aspartate phosphorylation: two-component systems and the limits of homology. *Trends Biochem Sci* 19: 485 – 490

van der Voorn L, Gebbink M, Plasterk A, Ploegh HL (1990) Characterization of a G-protein β-subunit gene from the nematode *Caenorhabditis elegans*. *J Mol Biol* 213: 17 – 26

Wang B, Kundu M (2017) Canonical and noncanonical functions of ULK/Atg1. *Curr Opin Cell Biol* 45: 47 – 54

Wieland T, Hippe HJ, Ludwig K, Zhou XB, Korth M, Klumpp S (2010) Reversible histidine phosphorylation in mammalian cells: a teeter-totter formed by nucleoside diphosphate kinase and protein histidine phosphatase 1. *Methods Enzymol* 471: 379 – 402

Yan D, Wu Z, Chisholm AD, Jin Y (2009) The DLK-1 kinase promotes mRNA stability and local translation in *C. elegans* synapses and axon regeneration. *Cell* 138: 1005 – 1018

License: This is an open access article under the terms of the Creative Commons Attribution License, which permits use, distribution and reproduction in any medium, provided the original work is properly cited.

Expanded View Figures

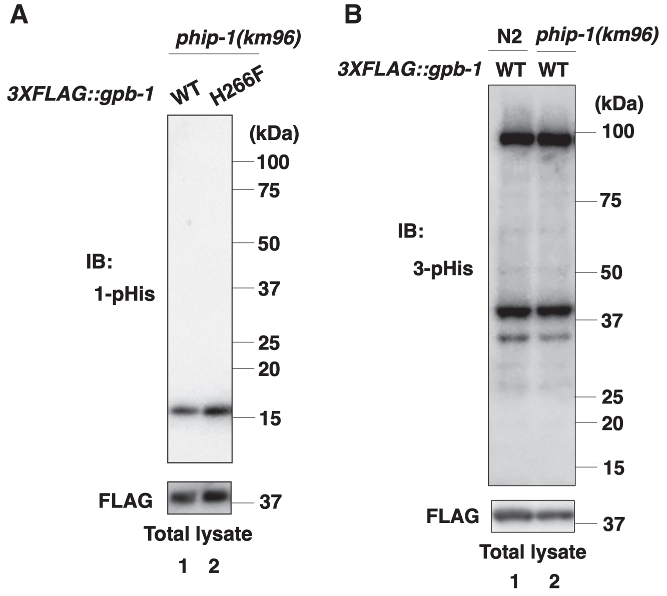

Figure EV1. His-phosphorylation in animals.

A 1-pHis levels in animals. The *phip-1(km96)* mutant animals carrying the *3XFLAG::gpb-1* or *3XFLAG::gpb-1(H266F)* knock-in allele were lysed. The lysates were immunoblotted (IB) with anti-1-pHis and anti-FLAG antibodies.

B The effect of the *phip-1(km96)* mutation on 3-pHis levels in animals. Wild-type N2 or *phip-1(km96)* mutant animals carrying the *3XFLAG::gpb-1* knock-in allele were lysed. The animal lysates were immunoblotted (IB) with anti-3-pHis and anti-FLAG antibodies.

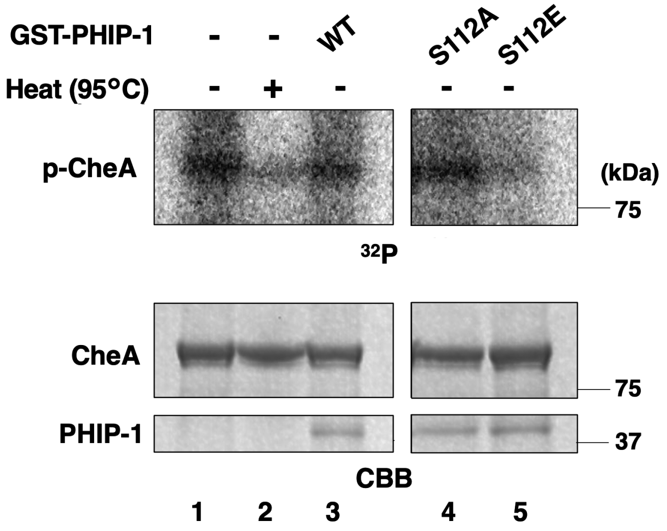

Figure EV2. Dephosphorylation of CheA by PHIP-1 *in vitro*.

GST-CheA was first incubated without GST-PHIP-1 for autophosphorylation. A-utophosphorylated CheA was then equally aliquoted and subjected to the *in vitro* phosphatase assay with GST-PHIP-1 or its variants. Phosphorylated CheA was detected by autoradiography. A heated sample (95°C) was used as a negative control. Protein input was confirmed by Coomassie Brilliant Blue (CBB) staining.

© 2022 The Authors

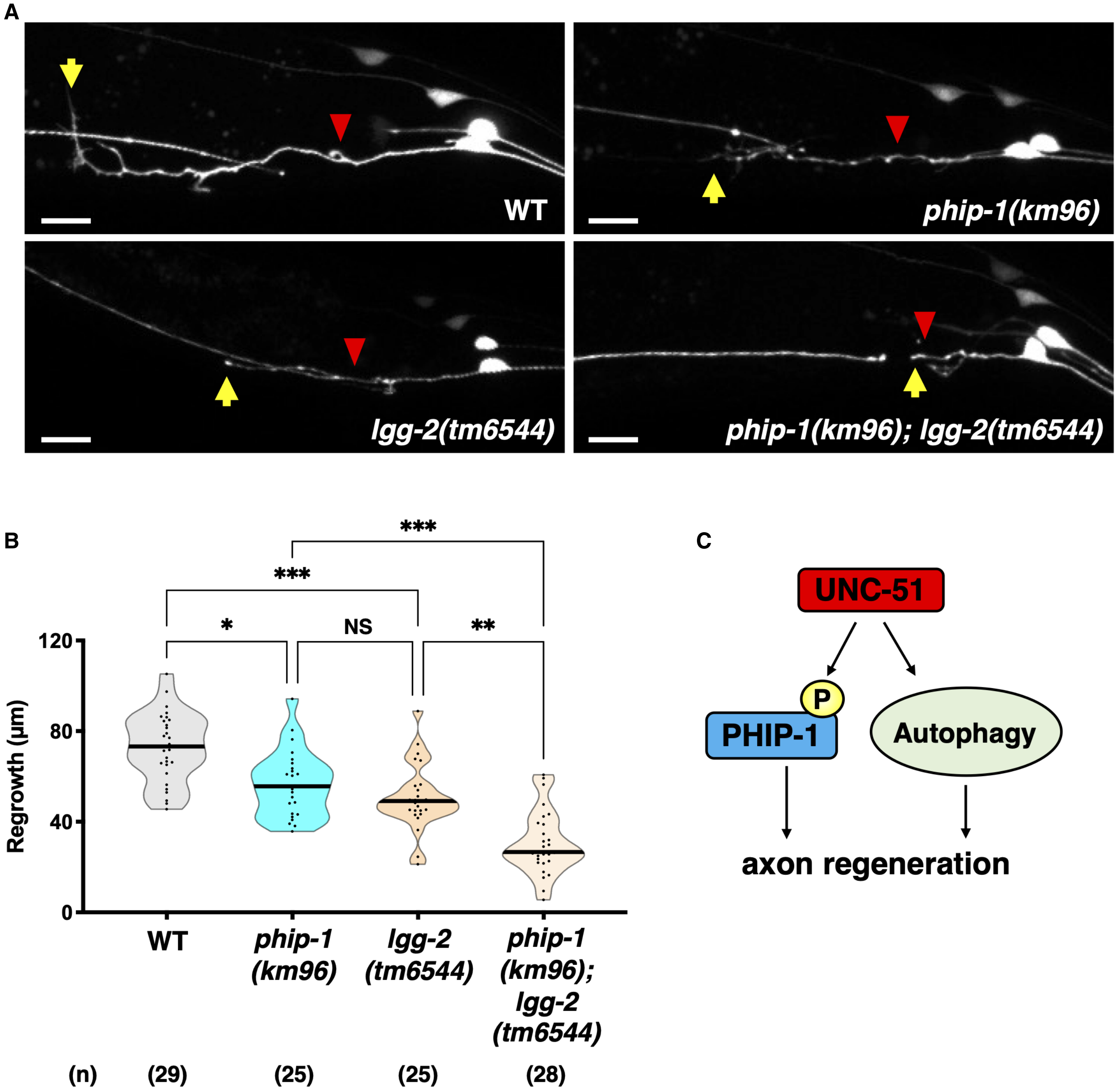

Figure EV3. UNC-51 regulates axon regeneration via PHIP-1 and autophagy.

A Representative PLM sensory neurons in indicated genotypes 24 h after laser surgery. Red arrowheads indicate cut sites. Yellow arrows indicate the tip of axotomized axons. Scale bar, 10 µm.

B Length of PLM regrowth 24 h after laser surgery. The number (*n*) of axons examined from two biological replicates is indicated. The black bar in each violin plot indicates the median. *$P < 0.05$, **$P < 0.01$, ***$P < 0.001$, as determined by the Kruskal–Wallis test and Dunn's multiple comparison test. NS, not significant.

C Downstream targets of UNC-51. UNC-51 promotes axon regeneration via phosphorylation of PHIP-1 and autophagy.

© 2022 The Authors
