# Supplementary material for: Histidine dephosphorylation of the Gβ protein GPB‐1 promotes axon regeneration in C. elegans
Source: EMBO Rep. 2022 Oct 24;23(12):e55076. doi: 10.15252/embr.202255076 (PMC9724660; doi:10.15252/embr.202255076)
Supplement: Supplementary file 4 [file EMBR-23-e55076-s002.pdf]

EMBO reports

# Histidine dephosphorylation of the Gβ protein GPB-1 promotes axon regeneration in *C. elegans*

Yoshiki Sakai, Hiroshi Hanafusa, Naoki Hisamoto, and Kunihiro Matsumoto
**DOI: 10.15252/embr.202255076**

*Corresponding author(s): Kunihiro Matsumoto (g44177a@cc.nagoya-u.ac.jp) , Naoki Hisamoto (i45556a@nucc.cc.nagoya-u.ac.jp)*



## Review Timeline:

| | |
|---|---|
| Submission Date: | 18th Mar 22 |
| Editorial Decision: | 30th May 22 |
| Revision Received: | 20th Aug 22 |
| Editorial Decision: | 28th Sep 22 |
| Revision Received: | 30th Sep 22 |
| Accepted: | 4th Oct 22 |

*Editor: Esther Schnapp*


## Transaction Report:

(Note: With the exception of the correction of typographical or spelling errors that could be a source of ambiguity, letters and reports are not edited. Depending on transfer agreements, referee reports obtained elsewhere may or may not be included in this compilation. Referee reports are anonymous unless the Referee chooses to sign their reports.)

**1st Editorial Decision** **30th May 2022**

Dear Dr. Matsumoto

Thank you for the submission of your manuscript to EMBO reports. We have now received the full set of referee reports that is pasted below.

As you will see, the referees acknowledge that the findings are potentially interesting. However, they also have several suggestions for how the data should be strengthened and the study improved. I think all points raised are reasonable and should be addressed. Please let me know in case you disagree, and we can discuss the revisions further, also in a video chat if you like.

I would thus like to invite you to revise your manuscript with the understanding that the referee concerns must be fully addressed and their suggestions taken on board. Please address all referee concerns in a complete point-by-point response. Acceptance of the manuscript will depend on a positive outcome of a second round of review. It is EMBO reports policy to allow a single round of major revision only and acceptance or rejection of the manuscript will therefore depend on the completeness of your responses included in the next, final version of the manuscript.

We realize that it is difficult to revise to a specific deadline. In the interest of protecting the conceptual advance provided by the work, we recommend a revision within 3 months (30th Aug 2022). Please discuss the revision progress ahead of this time with the editor if you require more time to complete the revisions.

You can either publish the study as a short report or as a full article. For short reports, the revised manuscript should not exceed 27,000 characters (including spaces but excluding materials & methods and references) and 5 main plus 5 expanded view figures. The results and discussion sections must further be combined, which will help to shorten the manuscript text by eliminating some redundancy that is inevitable when discussing the same experiments twice. For a normal article there are no length limitations, but it should have more than 5 main figures and the results and discussion sections must be separate. In both cases, the entire materials and methods must be included in the main manuscript file.

IMPORTANT NOTE: we perform an initial quality control of all revised manuscripts before re-review. Your manuscript will FAIL this control and the handling will be DELAYED if the following APPLIES:
1) A data availability section providing access to data deposited in public databases is missing. If you have not deposited any data, please add a sentence to the data availability section that explains that.
2) Your manuscript contains statistics and error bars based on n=2. Please use scatter blots in these cases. No statistics should be calculated if n=2.

When submitting your revised manuscript, please carefully review the instructions that follow below. Failure to include requested items will delay the evaluation of your revision.

1) a .docx formatted version of the manuscript text (including legends for main figures, EV figures and tables). Please make sure that the changes are highlighted to be clearly visible.

2) individual production quality figure files as .eps, .tif, .jpg (one file per figure). See https://wol-prod-cdn.literatumonline.com/pb-assets/embo-site/EMBOPress_Figure_Guidelines_061115-1561436025777.pdf for more info on how to prepare your figures.

3) We replaced Supplementary Information with Expanded View (EV) Figures and Tables that are collapsible/expandable online. A maximum of 5 EV Figures can be typeset. EV Figures should be cited as 'Figure EV1, Figure EV2" etc... in the text and their respective legends should be included in the main text after the legends of regular figures.

- For the figures that you do NOT wish to display as Expanded View figures, they should be bundled together with their legends in a single PDF file called *Appendix*, which should start with a short Table of Content. Appendix figures should be referred to in the main text as: "Appendix Figure S1, Appendix Figure S2" etc. See detailed instructions regarding expanded view here: <https://www.embopress.org/page/journal/14693178/authorguide#expandedview>

- Additional Tables/Datasets should be labeled and referred to as Table EV1, Dataset EV1, etc. Legends have to be provided in a separate tab in case of .xls files. Alternatively, the legend can be supplied as a separate text file (README) and zipped together with the Table/Dataset file.

4) a .docx formatted letter INCLUDING the reviewers' reports and your detailed point-by-point responses to their comments. As part of the EMBO Press transparent editorial process, the point-by-point response is part of the Review Process File (RPF), which will be published alongside your paper.

5) a complete author checklist, which you can download from our author guidelines <https://www.embopress.org/page/journal/14693178/authorguide>. Please insert information in the checklist that is also

reflected in the manuscript. The completed author checklist will also be part of the RPF.

6) Please note that all corresponding authors are required to supply an ORCID ID for their name upon submission of a revised manuscript (<https://orcid.org/>). Please find instructions on how to link your ORCID ID to your account in our manuscript tracking system in our Author guidelines
<https://www.embopress.org/page/journal/14693178/authorguide#authorshipguidelines>

7) Before submitting your revision, primary datasets produced in this study need to be deposited in an appropriate public database (see https://www.embopress.org/page/journal/14693178/authorguide#datadeposition). Please remember to provide a reviewer password if the datasets are not yet public. The accession numbers and database should be listed in a formal "Data Availability" section placed after Materials & Method (see also https://www.embopress.org/page/journal/14693178/authorguide#datadeposition). Please note that the Data Availability Section is restricted to new primary data that are part of this study. * Note - All links should resolve to a page where the data can be accessed. *
If your study has not produced novel datasets, please mention this fact in the Data Availability Section.

8) We would also encourage you to include the source data for figure panels that show essential data. Numerical data should be provided as individual .xls or .csv files (including a tab describing the data). For blots or microscopy, uncropped images should be submitted (using a zip archive if multiple images need to be supplied for one panel). Additional information on source data and instruction on how to label the files are available at
<https://www.embopress.org/page/journal/14693178/authorguide#sourcedata>.

9) Our journal also encourages inclusion of *data citations in the reference list* to directly cite datasets that were re-used and obtained from public databases. Data citations in the article text are distinct from normal bibliographical citations and should directly link to the database records from which the data can be accessed. In the main text, data citations are formatted as follows: "Data ref: Smith et al, 2001" or "Data ref: NCBI Sequence Read Archive PRJNA342805, 2017". In the Reference list, data citations must be labeled with "[DATASET]". A data reference must provide the database name, accession number/identifiers and a resolvable link to the landing page from which the data can be accessed at the end of the reference. Further instructions are available at https://www.embopress.org/page/journal/14693178/authorguide#referencesformat

10) Regarding data quantification (see Figure Legends:
https://www.embopress.org/page/journal/14693178/authorguide#figureformat)

The following points must be specified in each figure legend:

- the name of the statistical test used to generate error bars and P values,

- the number (n) of independent experiments (please specify technical or biological replicates) underlying each data point,

- the nature of the bars and error bars (s.d., s.e.m.),

- If the data are obtained from n {less than or equal to} 2, use scatter blots showing the individual data points.

Discussion of statistical methodology can be reported in the materials and methods section, but figure legends should contain a basic description of n, P and the test applied.

- Please also include scale bars in all microscopy images.

11) The journal requires a statement specifying whether or not authors have competing interests (defined as all potential or actual interests that could be perceived to influence the presentation or interpretation of an article). In case of competing interests, this must be specified in your disclosure statement. Further information: https://www.embopress.org/competing-interests

We would also welcome the submission of cover suggestions, or motifs to be used by our Graphics Illustrator in designing a cover.

As part of the EMBO publication's Transparent Editorial Process, EMBO reports publishes online a Review Process File (RPF) to accompany accepted manuscripts. This File will be published in conjunction with your paper and will include the referee reports, your point-by-point response and all pertinent correspondence relating to the manuscript.

You are able to opt out of this by letting the editorial office know (emboreports@embo.org). If you do opt out, the Review Process File link will point to the following statement: "No Review Process File is available with this article, as the authors have chosen not to make the review process public in this case."

I look forward to seeing a revised form of your manuscript when it is ready. Please use this link to submit your revision: https://embor.msubmit.net/cgi-bin/main.plex

Best regards,
Esther

Esther Schnapp, PhD
Senior Editor
EMBO reports

Referee #1:

Histidine phosphorylation is an non-canonical protein phosphorylation in animals. The biological significance of His-Phosphorylation is rather unclear. Recently the protein histidine phosphatase (PHPT1) and histidine kinase (NDK-1) is identified in mammals. In this paper author have provided genetic evidence that PHTP-1 and NDK-1 regulates axon regeneration in motor neuron model by regulating the histidine phosphorylation of GPB-1 Gb at His-266. Their data suggests that the His-phosphorylation plays an anti-regenerative role.
Further they went on to show that the conserved autophagy kinase UNC-51/ULK phosphorylates PHIP-1 at Ser-112 and this phosphorylation is essential for PHIP-1-mediated axon regeneration. This is an interesting manuscript. However there are some concern

1) Histidine phosphorylation is never demonstrated in C. elegans. The assumption of this study is that NDK-1 and PHTP-1 phosphorylates and dephosphorylates GBP1- at His-266 residue, respectively. It seems it's a big assumption. The authors needs to provide some evidence in worm system that Histidine-phosphorylation in the protein of their interest and that it is dependent on the NDK-1 and PHTP-1. Otherwise the data is mostly using the molecular genetics. If antibody against Histidine-phosphorylated amino acid is not available, they may use mass spectrometry.

2) A major role of UNC-51 is axon development, comes from many neuro-developmental studies. They are proposing that unc-51 is essential in axon regrowth using touch neuron system. I am aware that unc-51 mutant worms have very short PLM/ALM axons. How they rule out the possibility that effect in axon regeneration is not related to its initial axon developmental role? Also it is not clear why the authors while experimenting on the role of unc-51 in regulation of PHTP-1 moved to the touch neuron system from the motor neuron. Initially they were describing the role of NDK and PHTP using motor neuron system.

3) A general suggestion in the axon regeneration quantification: It will be good to show the length of regrowth as well.

4) It will be good to disclose the developmental phenotype (if any) of the mutants the authors dealt with in this manuscript

Referee #2:

This is an interesting paper whereby Sakai et al. demonstrate that reversible phosphorylation of H266 on GPB-1 G by NDPK and PHIP-1 regulate axonal regeneration via the regulation of GOA-1 Go signaling. The genetic evidence supporting this is convincing and novel and reaffirms genetically and in an in vivo model of axonal regeneration previous findings in mammalian cells.

- Given the availability of pHis antibodies, it would be nice for the authors to also demonstrate changes in H266 phosphorylation with these antibodies biochemically by immunoblotting with 1 and 3-pHis antibodies. This would also provide insight into whether GPB-1 G is histidine phosphorylated at the 1 or 3 position and which position is dephosphorylated by PHIP-1, of which little is currently known.

In order to try and understand how this is regulated molecularly, the authors identified UNC-51/ULK as a PHIP-1 interacting protein and demonstrate genetically that UNC-51 phosphorylation of Ser-112 is essential for PHIP-1 mediated axonal regeneration. While this data is convincing, the authors have an opportunity to perform additional experiments that may provide insight into other functions of PHIP-1 as well as the mechanism whereby phosphorylation of Ser-112 regulates PHIP-1. Specifically:
- The authors should more finely map the interaction of PHIP-1 to UNC-51. The initial screen identified AA 274-856 as the interacting protein. Have, the authors tried to identify a minimal sequence on UNC-51 that mediates binding? Is this sequence or interaction conserved with mammalian ULK 1 or 2? In addition, identifying a minimal sequence that mediates binding may

provide insight into identifying other proteins that may be regulated by PHIP-1.
- The authors propose that phosphorylation of Ser 112 regulates PHIP-1 function by affecting PHIP-1 localization. Do they have any evidence to support this? Have they tried to localize PHIP-1 in axons? How have they ruled out the possibility that phosphorylation does not regulate the catalytic activity of PHPT-1? This can be tested in an in vitro phosphatase assay.
- S112 is not conserved with mammalian PHPT1. It would be helpful for the authors to put this finding in the context with mammalian PHPT-1. Do they think this finding is unique to nematodes?

Minor point, PHPT-1 in mammalian cells have been shown to negatively regulate CD4 T cells via dephosphorylation of KCa3.1 (PNAS 2008:105(38): 14442-6, . This should be included in the discussion.

Referee #3:

The manuscript by Sakai et al. presents a detailed genetic analysis of how dynamic histidine phosphorylation regulates axon regeneration through GTP production. It is of interest because it reveals a new function for His-kinases in eukaryotes, which are not well studied, and adds to our understanding of how goa-1 is regulated in injured cells. The data mostly support the authors conclusions. I have relatively minor comments.

- Suppression of the unc-51 mutant by phip-1 is somewhat weak. This relationship should be revised in the text as crucial implies a stronger dependency than what is shown.

- Is it known how unc-51 regulate lgg-2? While the data show that lgg-2 functions in parallel to PHIP-1, it does not show that lgg-2 also functions downstream of unc-51. The authors should examine whether they do function in the same pathway.

- The conclusion that phip-1 and lgg-2 are partly responsible for unc-51 based regeneration depends on the answer to point 2. Is unc-51 function dependent on phip-1 and lgg-2?

- How GTP or unphosphorylated Gbeta is thought to regulate regeneration should be clarified in the discussion.

Dear Dr. Schnapp,

Enclosed please find our revised manuscript entitled: "Histidine phosphorylation-mediated signal transduction regulates axon regeneration in *Caenorhabditis elegans*" (by Sakai et al., EMBOR-2022-55076-T). Thank you for your e-mail of May 31, 2022, and the reviewers' comments on our manuscript. We appreciate these comments as they helped improve the manuscript. We have conducted experiments to address the comments of the reviewers. We believe that we have now satisfactorily addressed almost all concerns raised by the reviewers. We hope that this revised manuscript is now acceptable for publication in *EMBO Rep.* as *Article*. Our point-by-point responses to the reviewers' comments are provided below.

With best regards,

Kunihiro Matsumoto

Paper: EMBOR-2022-55076-T
Authors: Sakai et al.,
Title: Histidine phosphorylation-mediated signal transduction regulates axon regeneration in *Caenorhabditis elegans*

The manuscript has been revised in accordance with the comments given by the editor and three referees. Responses to the comments are as follows:

## Our responses to the comments of Editor

**1) A data availability section providing access to data deposited in public databases is missing. If you have not deposited any data, please add a sentence to the data availability section that explains that.**

We have added a statement in the Data availability section (p. 36, lines 738–739).

**2) Your manuscript contains statistics and error bars based on n=2. Please use scatter blots in these cases. No statistics should be calculated if n=2.**

We have conducted an additional aldicarb assay and replaced the data and figure legend (Appendix Fig. S2B).

## Our responses to the comments of Referee #1

**1) Histidine phosphorylation is never demonstrated in C. elegans. The assumption of this study is that NDK-1 and PHTP-1 phosphorylates and dephosphorylates GBP1- at His-266 residue, respectively. It seems it's a big assumption. The authors needs to provide some evidence in worm system that Histidine-phosphorylation in the protein of their interest and that it is dependent on the NDK-1 and PHTP-1. Otherwise the data is mostly using the molecular genetics. If antibody against Histidine-phosphorylated amino acid is not available, they may use mass spectrometry.**

As suggested, we have investigated His-phosphorylation of GPB-1 in the worm system.
pHis exists as two isomers, 1-pHis and 3-pHis, depending on the position of the phospho-acceptor nitrogen in the imidazole ring of histidine at positions, N1 and N3, respectively. Because the phosphoramidate (P–N) bond in pHis is thermally unstable, detecting pHis in biological samples is challenging. This problem has been largely solved with the development of monoclonal antibodies that specifically recognize 1-pHis or 3-pHis. We used these antibodies to evaluate His-phosphorylation in animals. To detect GPB-1, we tagged endogenous GPB-1 with 3XFLAG using CRISPR–Cas9-mediated genome editing (Appendix Fig. S1C).

Immunoblot signals for 1-pHis and 3-pHis detected one 1-pHis- and two or three 3-pHis-positive proteins in total lysates of *phip-1(km96)* mutant animals (Fig. 4A and Fig. EV1A and B). The 17-kDa band observed with

the 1-pHis antibody is likely to be NDK-1 (Fig. EV1A). Indeed, mammalian NDPK autophosphorylates at position N1 of His118, which is also conserved in NDK-1 (Fig. 1B).
When the lysate, dissolved in sample buffer, was heated at 95°C for 15 min prior to SDS-PAGE, the 3-pHis bands disappeared (Fig. 4A), indicating that the signals detected in the unheated sample are indeed 3-pHis proteins. Based on molecular weight analysis, the low molecular weight (37 kDa) 3-pHis protein corresponds to 3XFLAG::GPB-1. Consistently, the intensity of the 3-pHis signal was reduced in animals expressing the non-phosphorylatable FLAG::GPB-1(H266F) mutant protein (Fig. 4A). This result also suggests that GPB-1 has additional pHis site(s). Thus, PHIP-1 dephosphorylates GPB-1 pHis-266 in animals.

However, the intensity of the 3-pHis signals did not increase in *phip-1(km96)* mutants compared with wild-type (Fig EV1B), suggesting that under normal conditions, PHIP-1 is an inactive pHis-phosphatase. Consistently, we found that the PHIP-1 phosphatase activity is activated by UNC-51 phosphorylation (Fig. 4C and Fig. EV2).

To determine whether NDK-1 phosphorylates GPB-1 at His-266, an in vitro kinase assay was performed using purified recombinant glutathione *S*-transferase (GST)-tagged NDK-1. In mammalian cells, NDPK forms a complex with Gβγ and acts as a His-kinase for Gβ. Therefore, we used the *C. elegans* Gβγ complex as a substrate. Because *C. elegans* has two Gγ subunits, namely, GPC-1 and GPC-2, and GPC-2 works with GPB-1 in *C. elegans*, we used GPC-2 for the in vitro kinase assay. We co-expressed HA-tagged GPB-1 and T7-tagged GPC-2 in mammalian COS-7 cells. The GPB-1–GPC-2 complex was then immunopurified with anti-HA antibodies and incubated with GST-NDK-1 in vitro. GPB-1 phosphorylation was detected with anti-3-pHis antibodies. We found that NDK-1 phosphorylated GPB-1 and that the phosphorylation of GPB-1(H266F) by NDK-1 was reduced but not eliminated (Fig. 4B). These results support the possibility that NDK-1 phosphorylates multiple His-sites, including His-266, in GPB-1.

Furthermore, we demonstrated that PHIP-1 dephosphorylates pHis-GPB-1 in vitro using recombinant GST-tagged PHIP-1 proteins (Fig. 4C).

**2) A major role of UNC-51 is axon development, comes from many neuro-developmental studies. They are proposing that unc-51 is essential in axon regrowth using touch neuron system. I am aware that unc-51 mutant worms have very short PLM/ALM axons. How they rule out the possibility that effect in axon regeneration is not related to its initial axon developmental role? Also it is not clear why the authors while experimenting on the role of unc-51 in regulation of PHTP-1 moved to the touch neuron system from the motor neuron. Initially they were describing the role of NDK and PHTP using motor neuron system.**

We first examined whether UNC-51 is required for axon regeneration in D-type motor neurons. However, the loss-of-function *unc-51* mutation was

reported to severely affect the development of GABAergic D-type motor neurons (Appendix Fig. S4A). Therefore, it is difficult to assess the effect of *unc-51(ks49)* on axon regeneration of D-type neurons. By contrast, the *unc-51* mutation has only a weak effect on axon elongation along the anterior–posterior axis of touch sensory PLM neurons (Appendix Fig. S4B). We confirmed that *unc-51(ks49)* mutants displayed impaired axon regeneration in PLM neurons (Fig. 7A–C).

**3) A general suggestion in the axon regeneration quantification: It will be good to show the length of regrowth as well.**

As suggested, we have shown the length of axon regrowth in wild-type and *phip-1* mutants (Appendix Fig. S3).

**4) It will be good to disclose the developmental phenotype (if any) of the mutants the authors dealt with in this manuscript.**

The *phip-1* deletion mutants did not show any developmental phenotype.

**<u>Our responses to the comments of Referee #2</u>**

**- Given the availability of pHis antibodies, it would be nice for the authors to also demonstrate changes in H266 phosphorylation with these antibodies biochemically by immunoblotting with 1 and 3-pHis antibodies. This would also provide insight into whether GPB-1 Gβ is histidine phosphorylated at the 1 or 3 position and which position is dephosphorylated by PHIP-1, of which little is currently known.**

As suggested, we demonstrated changes in GPB-1 His-266 phosphorylation using 1-pHis and 3-pHis antibodies.

To detect GPB-1 protein in animals, we inserted the 3XFLAG tag into the N-terminus of the endogenous *gpb-1* locus with the CRISPR–Cas9 method (Appendix Fig. S1C).

Immunoblot signals for 1-pHis and 3-pHis detected one 1-pHis- protein and two or three 3-pHis-positive proteins in total lysates of animals (Fig. 4A and Fig. EV1A and B). The 17-kDa band observed with the 1-pHis antibody is likely to be NDK-1 (Fig. EV1A). Indeed, mammalian NDPK autophosphorylates at position N1 of His118, which is also conserved in NDK-1 (Fig. 1B).
Based on molecular weight analysis, the low molecular weight (37 kDa) 3-pHis protein corresponds to 3XFLAG::GPB-1. Consistently, the intensity of the 3-pHis signal was reduced in animals expressing the non-phosphorylatable FLAG::GPB-1(H266F) mutant protein (Fig. 4A).

**In order to try and understand how this is regulated molecularly, the authors identified UNC-51/ULK as a PHIP-1 interacting protein and demonstrate**

**genetically that UNC-51 phosphorylation of Ser-112 is essential for PHIP-1 mediated axonal regeneration. While this data is convincing, the authors have an opportunity to perform additional experiments that may provide insight into other functions of PHIP-1 as well as the mechanism whereby phosphorylation of Ser-112 regulates PHIP-1.**

**Specifically:**
**- The authors should more finely map the interaction of PHIP-1 to UNC-51. The initial screen identified AA 274-856 as the interacting protein. Have, the authors tried to identify a minimal sequence on UNC-51 that mediates binding? Is this sequence or interaction conserved with mammalian ULK 1 or 2? In addition, identifying a minimal sequence that mediates binding may provide insight into identifying other proteins that may be regulated by PHIP-1.**

We did not attempt to identify the minimal sequence mediating UNC-51–PHPT-1 binding.

We examined the relationship between ULK/UNC-51 and PHPT1/PHIP-1 and found that mammalian ULK1 does not phosphorylate PHPT1 (Appendix Fig S5A). Therefore, the relationship between ULK1 and PHPT1 is unlikely to be conserved.

To search for specific PHIP-1 substrates, a catalytically dead mutant form of PHIP-1 is useful as a substrate-trapping mutant, an approach that has been exploited successfully for identifying substrates of PTPs. To identify targets of PHIP-1, we performed a yeast two-hybrid screen using the phosphatase-negative PHIP-1(H45A) as bait to isolate proteins that interact with PHIP-1. We isolated GPB-1 Gβ and GPD-2/GPD-3/GPD-4, glyceraldehyde-3-phosphate dehydrogenase (GAPDH) (Fig. 3A and B and Appendix Table S1). Because mammalian GNB1 Gβ and GAPDH are known pHis proteins, GPB-1 and GPD-2/GPD-3/GPD-4 are candidate targets for PHIP-1. Thus, two-hybrid screening with PHPT-1(H45A) is useful for identifying PHIP-1 targets.

**-The authors propose that phosphorylation of Ser 112 regulates PHIP-1 function by affecting PHIP-1 localization. Do they have any evidence to support this? Have they tried to localize PHIP-1 in axons? How have they ruled out the possibility that phosphorylation does not regulate the catalytic activity of PHPT-1? This can be tested in an in vitro phosphatase assay.**

As suggested, we tested the effect of PHIP-1 Ser-112 phosphorylation on the catalytic activity of PHIP-1. We showed that PHIP-1(S112E) efficiently dephosphorylated pHis-GPB-1 in vitro, whereas PHIP-1(S112A) did not (Fig. 4C). These results indicate that phosphorylation of Ser-112 in PHIP-1 activates its phosphatase activity. We further confirmed this possibility using recombinant GST-tagged bacterial CheA, which autophosphorylates itself on histidine. We demonstrated that wild-type PHIP-1 weakly dephosphorylated pHis-CheA, whereas PHIP-1(S112E) exhibited stronger phosphatase activity than

wild-type and PHIP-1(S112A) (Fig EV2). Thus, UNC-51 phosphorylation activates the catalytic activity of PHIP-1.

**-S112 is not conserved with mammalian PHPT1. It would be helpful for the authors to put this finding in the context with mammalian PHPT-1. Do they think this finding is unique to nematodes?**

We examined whether the relationship between UNC-51 and PHIP-1 is functionally conserved in mammals. We showed that GST-ULK1 phosphorylated itself but not PHPT1 (Appendix Fig S5A). Consistently, Ser-112 in PHIP-1 corresponds with Ala-121 in PHPT1, but this site is not conserved (Appendix Fig S5B). However, the region surrounding Ser-112 in PHIP-1 is highly conserved with the corresponding region in PHPT1, and the Thr-119 residue is present in PHPT1 (Appendix Fig S5B). These findings raise the possibility that an unknown kinase activates PHPT1 by phosphorylating PHPT1 on Thr-119.

**Minor point, PHPT-1 in mammalian cells have been shown to negatively regulate CD4 T cells via dephosphorylation of KCa3.1 (PNAS 2008:105(38): 14442-6. This should be included in the discussion.**

As suggested, this reference is included in the Discussion section (p. 22, lines 422–424).

**<u>Our responses to the comments of Referee #3</u>**

**Relatively minor comments.**

**- Suppression of the unc-51 mutant by phip-1 is somewhat weak. This relationship should be revised in the text as crucial implies a stronger dependency than what is shown.**

We have made suitable revisions (p. 17, lines 345–346).

**- Is it known how unc-51 regulate lgg-2? While the data show that lgg-2 functions in parallel to PHIP-1, it does not show that lgg-2 also functions downstream of unc-51. The authors should examine whether they do function in the same pathway.**

ULK kinase and LC3 are components of the primary autophagy machinery and are involved in the initiation of autophagosome biogenesis in mammals. Thus, ULK/UNC-51 and LC3/LGG-2 act in the same pathway in autophagy.

In addition, Crawley et al. showed that UNC-51 and LGG-2 regulate axon termination and synapse maintenance through autophagosome formation in axonal compartments (Nat. Commun. 10, 5017, 2019). In this pathway, LGG-2 functions downstream of UNC-51.

**- The conclusion that phip-1 and lgg-2 are partly responsible for unc-51 based regeneration depends on the answer to point 2. Is unc-51 function dependent on phip-1 and lgg-2?**

Because the *phip-1* mutation does not affect autophagy, it is unlikely that UNC-51 function is dependent on PHIP-1.

Crawley et al. showed that UNC-51 and LGG-2 regulate axon termination and synapse maintenance through autophagosome formation in axonal compartments (Nat. Commun. 10, 5017, 2019). In this pathway, LGG-2 functions downstream of UNC-51.

**- How GTP or unphosphorylated Gbeta is thought to regulate regeneration should be clarified in the discussion.**

In *C. elegans*, two different Gα subunits, namely, EGL-30 Gqα and GOA-1 Goα participate in axon regeneration (Fig. 5C). EGL-30 activates phospholipase Cβ (PLCβ) EGL-8, which in turn generates DAG, resulting in the activation of the protein kinase C (PKC) homolog TPA-1. TPA-1 promotes axon regeneration by activating the JNK MAP kinase (MAPK) cascade. GTP-bound GOA-1 antagonizes the EGL-30 signaling cascade and inhibits axon regeneration. This inhibition is mediated by His-266 phosphorylation of GPB-1 Gβ, which leads to activation of GOA-1 Goα signaling. Thus, these findings suggest a link between the GPCR-independent activation of Goα and His-phosphorylation of Gβ in the regulation of axon regeneration. We have mentioned this in p. 15, lines 272–282.

Dear Dr. Matsumoto

Thank you for the submission of your revised manuscript. We have now received the enclosed referee reports, and I am happy to say that all referees support its publication now. Only a few editorial requests will need to be addressed before we can proceed with the official acceptance of your manuscript.

- Please correct the conflict of interest subheading to "Disclosure and Competing Interest Statement "

- Please remove the author credits from the ms file. We now use CRediT to specify the contributions of each author in the journal submission system. CRediT replaces the author contribution section. Please use the free text box to provide more detailed descriptions, if you wish. See also guide to authors https://www.embopress.org/page/journal/14693178/authorguide#authorshipguidelines.

- Please add the FUNDING INFO: Grant # 21J15589 to the ms file.

- Appendix Table S3 callout is missing in the ms text, please add.

- Please add the subheading 'Expanded View Figure Legends'.

I would like to suggest some changes to the title and abstract that needs to be written in present tense. Please let me know whether you agree with the following and whether all statements are correct:

Histidine dephosphorylation of GPB-1 promotes axon regeneration in C. elegans

Histidine phosphorylation is an emerging non-canonical protein phosphorylation in animals, yet its physiological role remains largely unexplored. The protein histidine phosphatase (PHPT1) was recently identified for the first time in mammals. Here we report that PHIP-1, an ortholog of PHPT1 in Caenorhabditis elegans, promotes axon regeneration by dephosphorylating GPB-1 Gβ at His-266 and inactivating GOA-1 Goα signaling, a negative regulator of axon regeneration. Overexpression of the histidine kinase NDK-1 also inhibits axon regeneration via GPB-1 His-266 phosphorylation. Thus, His-phosphorylation plays an anti-regenerative role in C. elegans. Furthermore, we identify a conserved UNC-51/ULK kinase that functions in autophagy as a PHIP-1-binding protein. We demonstrate that UNC-51 phosphorylates PHIP-1 at Ser-112 and activates its catalytic activity, and that this phosphorylation is required for PHIP-1-mediated axon regeneration. This study reveals a molecular link from ULK to protein histidine phosphatase, which facilitates axon regeneration by inhibiting trimeric G protein signaling.

EMBO press papers are accompanied online by A) a short (1-2 sentences) summary of the findings and their significance, B) 2-3 bullet points highlighting key results and C) a synopsis image that is exactly 550 pixels wide and 200-600 pixels high (the height is variable). You can either show a model or key data in the synopsis image. Please note that text needs to be readable at the final size. Please send us this information along with the revised manuscript.

I look forward to seeing a final version of your manuscript as soon as possible. Please use this link to submit your revision: https://embor.msubmit.net/cgi-bin/main.plex

Best regards,
Esther

Esther Schnapp, PhD
Senior Editor
EMBO reports

Referee #1:

The authors have addressed all the comments raised by me. The manuscripts has significantly improved. Therefore, it is suitable for publication in EMBO reports

Referee #2:

the authors have successfully answered the questions proposed by the reviewers

Referee #3:

The authors have addressed my comments.

The authors have addressed all minor editorial requests.

Dr. Kunihiro Matsumoto
Nagoya University
Chikusa-ku
Nagoya, Aichi 464-8602
Japan

Dear Dr. Matsumoto,

I am very pleased to accept your manuscript for publication in the next available issue of EMBO reports. Thank you for your contribution to our journal.

At the end of this email I include important information about how to proceed. Please ensure that you take the time to read the information and complete and return the necessary forms to allow us to publish your manuscript as quickly as possible.

As part of the EMBO publication's Transparent Editorial Process, EMBO reports publishes online a Review Process File to accompany accepted manuscripts. As you are aware, this File will be published in conjunction with your paper and will include the referee reports, your point-by-point response and all pertinent correspondence relating to the manuscript.

If you do NOT want this File to be published, please inform the editorial office within 2 days, if you have not done so already, otherwise the File will be published by default [contact: emboreports@embo.org]. If you do opt out, the Review Process File link will point to the following statement: "No Review Process File is available with this article, as the authors have chosen not to make the review process public in this case."

Should you be planning a Press Release on your article, please get in contact with emboreports@wiley.com as early as possible, in order to coordinate publication and release dates.

Thank you again for your contribution to EMBO reports and congratulations on a successful publication. Please consider us again in the future for your most exciting work.

Yours sincerely,

Esther Schnapp, PhD
Senior Editor
EMBO reports

***********************************************************************************

THINGS TO DO NOW:

Please note that you will be contacted by Wiley Author Services to complete licensing and payment information. The required 'Page Charges Authorization Form' is available here: https://www.embopress.org/pb-assets/embo-site/er_apc.pdf - please download and complete the form and return to embopressproduction@wiley.com

EMBO Press participates in many Publish and Read agreements that allow authors to publish Open Access with reduced/no publication charges. Check your eligibility: https://authorservices.wiley.com/author-resources/Journal-Authors/open-access/affiliation-policies-payments/index.html

You will receive proofs by e-mail approximately 2-3 weeks after all relevant files have been sent to our Production Office; you should return your corrections within 2 days of receiving the proofs.

Please inform us if there is likely to be any difficulty in reaching you at the above address at that time. Failure to meet our deadlines may result in a delay of publication, or publication without your corrections.

All further communications concerning your paper should quote reference number EMBOR-2022-55076V3 and be addressed to emboreports@wiley.com.

Should you be planning a Press Release on your article, please get in contact with emboreports@wiley.com as early as possible, in order to coordinate publication and release dates.

## EMBO Press Author Checklist

| | |
|---|---|
| Corresponding Author Name: Kunihiro Matsumoto | |
| Journal Submitted to: EMBO Reports | |
| Manuscript Number: 2022-55076V2 | |

**USEFUL LINKS FOR COMPLETING THIS FORM**
The EMBO Journal - Author Guidelines
EMBO Reports - Author Guidelines
Molecular Systems Biology - Author Guidelines
EMBO Molecular Medicine - Author Guidelines

**Reporting Checklist for Life Science Articles (updated January 2022)**
This checklist is adapted from Materials Design Analysis Reporting (MDAR) Checklist for Authors. MDAR establishes a minimum set of requirements in transparent reporting in the life sciences (see Statement of Task: 10.31222/osf.io/9sm4x). Please follow the journal's guidelines in preparing your manuscript.
**Please note that a copy of this checklist will be published alongside your article.**

**Abridged guidelines for figures**
**1. Data**
The data shown in figures should satisfy the following conditions:
- the data were obtained and processed according to the field's best practice and are presented to reflect the results of the experiments in an accurate and unbiased manner.
- ideally, figure panels should include only measurements that are directly comparable to each other and obtained with the same assay.
- plots include clearly labeled error bars for independent experiments and sample sizes. Unless justified, error bars should not be shown for technical replicates.
- if n<5, the individual data points from each experiment should be plotted. Any statistical test employed should be justified.
- Source Data should be included to report the data underlying figures according to the guidelines set out in the authorship guidelines on Data Presentation.

**2. Captions**
Each figure caption should contain the following information, for each panel where they are relevant:
- a specification of the experimental system investigated (eg cell line, species name).
- the assay(s) and method(s) used to carry out the reported observations and measurements.
- an explicit mention of the biological and chemical entity(ies) that are being measured.
- an explicit mention of the biological and chemical entity(ies) that are altered/varied/perturbed in a controlled manner.
- the exact sample size (n) for each experimental group/condition, given as a number, not a range;
- a description of the sample collection allowing the reader to understand whether the samples represent technical or biological replicates (including how many animals, litters, cultures, etc.).
- a statement of how many times the experiment shown was independently replicated in the laboratory.
- definitions of statistical methods and measures:
  - common tests, such as t-test (please specify whether paired vs. unpaired), simple χ2 tests, Wilcoxon and Mann-Whitney tests, can be unambiguously identified by name only, but more complex techniques should be described in the methods section;
  - are tests one-sided or two-sided?
  - are there adjustments for multiple comparisons?
  - exact statistical test results, e.g., P values = x but not P values < x;
  - definition of 'center values' as median or average;
  - definition of error bars as s.d. or s.e.m.

**Please complete ALL of the questions below.**
**Select "Not Applicable" only when the requested information is not relevant for your study.**

**Materials**

| Newly Created Materials | Information included in the manuscript? | In which section is the information available? (Reagents and Tools Table, Materials and Methods, Figures, Data Availability Section) |
|---|---|---|
| New materials and reagents need to be available; do any restrictions apply? | Not Applicable | |

| Antibodies | Information included in the manuscript? | In which section is the information available? (Reagents and Tools Table, Materials and Methods, Figures, Data Availability Section) |
|---|---|---|
| For **antibodies** provide the following information:<br>- Commercial antibodies: RRID (if possible) or supplier name, catalogue number and or/clone number<br>- Non-commercial: RRID or citation | Yes | Materials and Methods |

| DNA and RNA sequences | Information included in the manuscript? | In which section is the information available? (Reagents and Tools Table, Materials and Methods, Figures, Data Availability Section) |
|---|---|---|
| **Short novel DNA or RNA including primers, probes**: provide the sequences. | Yes | Appendix Table 3 |

| Cell materials | Information included in the manuscript? | In which section is the information available? (Reagents and Tools Table, Materials and Methods, Figures, Data Availability Section) |
|---|---|---|
| **Cell lines:** Provide species information, strain. Provide accession number in repository **OR** supplier name, catalog number, clone number, and/**OR** RRID. | Yes | Materials and Methods |
| **Primary cultures:** Provide species, strain, sex of origin, genetic modification status. | Not Applicable | |
| Report if the cell lines were recently **authenticated** (e.g., by STR profiling) and tested for mycoplasma contamination. | Not Applicable | |

| Experimental animals | Information included in the manuscript? | In which section is the information available? (Reagents and Tools Table, Materials and Methods, Figures, Data Availability Section) |
|---|---|---|
| **Laboratory animals or Model organisms:** Provide species, strain, sex, age, genetic modification status. Provide accession number in repository **OR** supplier name, catalog number, clone number, **OR** RRID. | Yes | Materials and Methods |
| **Animal observed in or captured from the field:** Provide species, sex, and age where possible. | Not Applicable | |
| Please detail **housing and husbandry conditions**. | Yes | Materials and Methods |

| Plants and microbes | Information included in the manuscript? | In which section is the information available? (Reagents and Tools Table, Materials and Methods, Figures, Data Availability Section) |
|---|---|---|
| **Plants:** provide species and strain, ecotype and cultivar where relevant, unique accession number if available, and source (including location for collected wild specimens). | Not Applicable | |
| **Microbes:** provide species and strain, unique accession number if available, and source. | Not Applicable | |

| Human research participants | Information included in the manuscript? | In which section is the information available? (Reagents and Tools Table, Materials and Methods, Figures, Data Availability Section) |
|---|---|---|
| If collected and within the bounds of privacy constraints report on age, sex and gender or ethnicity for all study participants. | Not Applicable | |

| Core facilities | Information included in the manuscript? | In which section is the information available? (Reagents and Tools Table, Materials and Methods, Figures, Data Availability Section) |
|---|---|---|
| If your work benefited from core facilities, was their service mentioned in the acknowledgments section? | Not Applicable | |

**Design**

| Study protocol | Information included in the manuscript? | In which section is the information available? (Reagents and Tools Table, Materials and Methods, Figures, Data Availability Section) |
|---|---|---|
| If study protocol has been **pre-registered, provide DOI in the manuscript**. For clinical trials, provide the trial registration number **OR** cite DOI. | Not Applicable | |
| Report the **clinical trial registration number** (at ClinicalTrials.gov or equivalent), where applicable. | Not Applicable | |

| Laboratory protocol | Information included in the manuscript? | In which section is the information available? (Reagents and Tools Table, Materials and Methods, Figures, Data Availability Section) |
|---|---|---|
| Provide DOI OR other citation details if **external detailed step-by-step protocols** are available. | Not Applicable | |

| Experimental study design and statistics | Information included in the manuscript? | In which section is the information available? (Reagents and Tools Table, Materials and Methods, Figures, Data Availability Section) |
|---|---|---|
| Include a statement about **sample size** estimate even if no statistical methods were used. | Yes | Materials and Methods |
| Were any steps taken to minimize the effects of subjective bias when allocating animals/samples to treatment (e.g. **randomization procedure**)? If yes, have they been described? | Yes | Materials and Methods |
| Include a statement about **blinding** even if no blinding was done. | Yes | Materials and Methods |
| Describe **inclusion/exclusion criteria** if samples or animals were excluded from the analysis. Were the criteria pre-established?<br><br>If sample or data points were omitted from analysis, report if this was due to attrition or intentional exclusion and provide justification. | Yes | Materials and Methods |
| For every figure, are **statistical tests** justified as appropriate? Do the data meet the assumptions of the tests (e.g., normal distribution)? Describe any methods used to assess it. Is there an estimate of variation within each group of data? Is the variance similar between the groups that are being statistically compared? | Yes | Materials and Methods |

| Sample definition and in-laboratory replication | Information included in the manuscript? | In which section is the information available? (Reagents and Tools Table, Materials and Methods, Figures, Data Availability Section) |
|---|---|---|
| In the figure legends: state number of times the experiment was **replicated** in laboratory. | Yes | Figure legends |
| In the figure legends: define whether data describe **technical or biological replicates**. | Yes | Figure legends |

## Ethics

| Ethics | Information included in the manuscript? | In which section is the information available? (Reagents and Tools Table, Materials and Methods, Figures, Data Availability Section) |
|---|---|---|
| Studies involving **human participants**: State details of **authority granting ethics approval** (IRB or equivalent committee(s), provide reference number for approval. | Not Applicable | |
| Studies involving **human participants**: Include a statement confirming that **informed consent** was obtained from all subjects and that the experiments conformed to the principles set out in the WMA Declaration of Helsinki and the Department of Health and Human Services Belmont Report. | Not Applicable | |
| Studies involving **human participants:** For publication of **patient photos**, include a statement confirming that consent to publish was obtained. | Not Applicable | |
| Studies involving experimental **animals**: State details of **authority granting ethics approval** (IRB or equivalent committee(s), provide reference number for approval. Include a statement of compliance with ethical regulations. | Not Applicable | |
| Studies involving **specimen and field samples:** State if relevant **permits** obtained, provide details of authority approving study; if none were required, explain why. | Not Applicable | |

| Dual Use Research of Concern (DURC) | Information included in the manuscript? | In which section is the information available? (Reagents and Tools Table, Materials and Methods, Figures, Data Availability Section) |
|---|---|---|
| Could your study fall under dual use research restrictions? Please check biosecurity documents and list of **select agents and toxins** (CDC): https://www.selectagents.gov/sat/list.htm | Not Applicable | |
| If you used a select agent, is the security level of the lab appropriate and reported in the manuscript? | Not Applicable | |
| If a study is subject to dual use research of concern regulations, is the name of the **authority granting approval and reference number** for the regulatory approval provided in the manuscript? | Not Applicable | |

## Reporting

The MDAR framework recommends adoption of discipline-specific guidelines, established and endorsed through community initiatives. Journals have their own policy about requiring specific guidelines and recommendations to complement MDAR.

| Adherence to community standards | Information included in the manuscript? | In which section is the information available? (Reagents and Tools Table, Materials and Methods, Figures, Data Availability Section) |
|---|---|---|
| State if relevant guidelines or checklists (e.g., **ICMJE, MIBBI, ARRIVE, PRISMA**) have been followed or provided. | Not Applicable | |
| For **tumor marker prognostic studies,** we recommend that you follow the **REMARK** reporting guidelines (see link list at top right). See author guidelines, under 'Reporting Guidelines'. Please confirm you have followed these guidelines. | Not Applicable | |
| For **phase II and III randomized controlled trials**, please refer to the **CONSORT** flow diagram (see link list at top right) and submit the CONSORT checklist (see link list at top right) with your submission. See author guidelines, under 'Reporting Guidelines'. Please confirm you have submitted this list. | Not Applicable | |

## Data Availability

| Data availability | Information included in the manuscript? | In which section is the information available? (Reagents and Tools Table, Materials and Methods, Figures, Data Availability Section) |
|---|---|---|
| Have **primary datasets** been deposited according to the journal's guidelines (see 'Data Deposition' section) and the respective accession numbers provided in the Data Availability Section? | Not Applicable | |
| Were **human clinical and genomic datasets** deposited in a public access-controlled repository in accordance to ethical obligations to the patients and to the applicable consent agreement? | Not Applicable | |
| Are **computational models** that are central and integral to a study available without restrictions in a machine-readable form? Were the relevant accession numbers or links provided? | Not Applicable | |
| If publicly available data were reused, provide the respective **data citations in the reference list**. | Not Applicable | |
